# Protocol for assessing protein expression levels and localization during fission yeast cytokinesis

**DOI:** 10.1016/j.xpro.2026.104839

**Published:** 2026-09-17

**Authors:** Jack R. Gregory, Yanfang Ye, Davinder Singh, Aysha H. Osmani, Jian-Qiu Wu

**Affiliations:** 1 Department of Molecular Genetics, The Ohio State University, Columbus, OH 43210, USA; 2 Cellular, Molecular, and Biochemical Sciences Program, The Ohio State University, Columbus, OH 43210, USA; 3 Graduate Program of Molecular, Cellular, and Developmental Biology, The Ohio State University, Columbus, OH 43210, USA; 4 Postdoctoral Candidate Pelotonia Scholars Program, The Ohio State University, Columbus, OH 43210, USA

**Keywords:** Cell Biology, Microscopy, Model Organisms

## Abstract

The fission yeast *Schizosaccharomyces pombe* is a genetically tractable model that shares mechanisms for cytokinesis with higher eukaryotes. Here, we present a protocol for analyzing fission yeast cytokinesis proteins. We include steps for gene targeting by homologous recombination, functionality tests by genetic interactions, and imaging by high-spatiotemporal resolution microscopy. Next, we describe procedures for image analyses using Fiji, protein interactions by co-immunoprecipitation, and measuring protein levels by Western blotting. These techniques are transferable to other subcellular locations and model organisms.

For complete details on the use and execution of this protocol, please refer to Ye et al.^1^

## Highlights

• Updated instructions for PCR-based gene targeting protocol for fission yeast
• Streamlined process for assessing protein localization, kinetics, and concentration
• Steps for easy-to-implement lyophilization of fission yeast for protein extraction

## Abstract (editor highlights)

Publisher’s note: Undertaking any experimental protocol requires adherence to local institutional guidelines for laboratory safety and ethics.

## Graphical abstract

**Figure:**
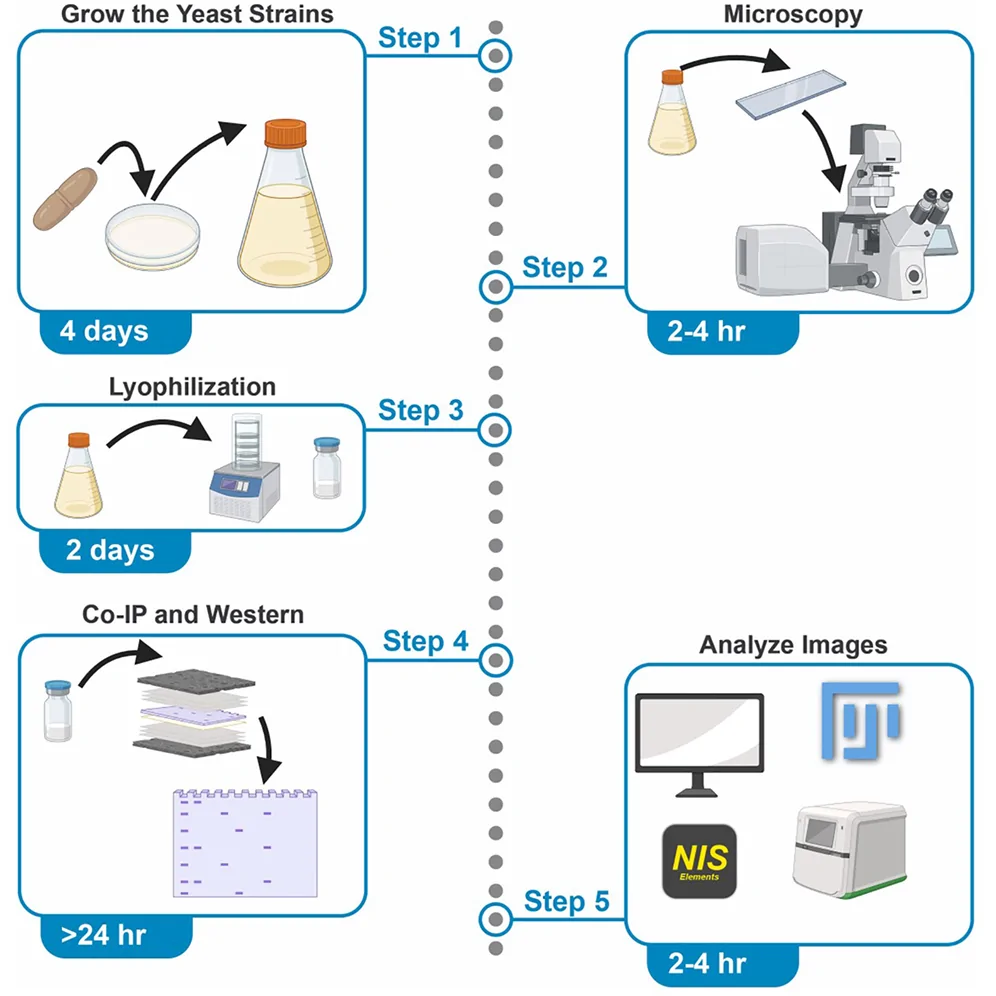

## Teaser

The fission yeast *Schizosaccharomyces pombe* is a genetically tractable model that shares mechanisms for cytokinesis with higher eukaryotes. Here, we present a protocol for analyzing fission yeast cytokinesis proteins. We include steps for gene targeting by homologous recombination, functionality tests by genetic interactions, and imaging by high-spatiotemporal microscopy. Next, we describe procedures for image analyses using Fiji, protein interactions by co-immunoprecipitation, and measuring protein levels by western blotting. These techniques are transferable to other subcellular locations and model organisms.

## Before you begin

The protocol below describes the processes of constructing strains; obtaining protein localization, intensity, and dynamics at the division site during cytokinesis; assessing protein interactions; and total protein levels in cell extracts in the fission yeast *Schizosaccharomyces pombe*. The techniques used here are transferable to other locations in the cell and other model systems.

### Innovation

This protocol has multiple advantages and innovations compared to the currently available methods. First, it contains a thorough step-by-step guide for PCR and homologous recombination-based gene targeting with updates and optimizations since the seminal fission yeast gene targeting method in 1998.^2^ We have optimized the protocol over nearly three decades across many publications.^3^^,^^4^^,^^5^^,^^6^^,^^7^^,^^8^^,^^9^^,^^10^^,^^11^^,^^12^^,^^13^^,^^14^ Primers with 70 instead of 80 nucleotides’ homology to the target gene are sufficient. More selectable markers and epitope tags, especially the monomeric fluorescent proteins, are successfully employed. We also emphasize the importance of rigorous functionality tests of fusion proteins and protein expression at native levels. Second, this protocol describes in detail the usages of fluorescence-intensity quantification and 3D image projections in *S. pombe*. These quantitative fluorescence microscopy techniques are successfully utilized in our publications to understand protein organization at the division site.^15^^,^^16^^,^^17^^,^^18^^,^^19^ Those analyses can easily be transferred to other sub-cellular locations and different organisms. Third, we describe how to obtain yeast cell extracts using lyophilized cells, which is efficient, straight-forward, reliable, and cost effective. No expensive equipment and reagents are needed to lyophilize cells. Lastly, our protocol combines the multiple advances into one seamless workflow. In summary, this workflow has the advantages of integrating optimized experimental designs and refined innovative methods into one easy to implement and modify protocol for a wide range of research areas.

### Strain creation by gene targeting and genetic crosses

**Timing: 1–2 weeks starting from receiving primers for gene targeting, and the duration dependent on the growth temperature and medium used**

1. Obtain primers for gene targeting.
  a. If a C-terminally tagged Protein of Interest (POI) is desired:
    i. Design the forward primer with 70 nucleotides (nt) of homology to the last 70 base pairs (bp) of the coding region for the POI gene, excluding the stop codon (since it will be included with the fluorescent protein sequence), and followed by 20 nt of homology to the pFA6a vectors (CGG ATC CCC GGG TTA ATT AA) upstream of the fluorescent protein.^2^
      **CRITICAL:** The stop codon cannot be included in the primer, otherwise, the epitope tag will not be expressed.
    ii. Design the reverse primer with 70 nt of complement to 3′UTR of the DNA sequences of POI, immediately downstream of the stop codon and followed by 20 nt (GAATTC GAGCTC GTTTAAAC) from the sequence downstream of the terminator of the selectable marker cassette.
  b. If an N-terminally tagged POI is desired:
    i. Design the forward primer with 70 nt of homology to the 5′ UTR immediately upstream of the start codon for the POI gene and followed by 20 nt of homology to the pFA6a vectors (GAATTC GAGCTC GTTTAAAC) from the sequence downstream of the terminator of the selectable marker cassette.^2^
    ii. Design the reverse primer with 70 nt of complement to the first 70 nt of the encoding DNA sequences of the POI including the start codon and followed by the complement to the last 20 nt of the encoding sequences for the fluorescent protein or other epitope tags.
      ***Note:*** The Jürg Bähler lab developed PPPP (Pombe PCR Primer Programs), multiple convenient Web-interface scripts that help design primers for PCR-based gene targeting in *S. pombe.*
      ***Note:*** This assumes that you have obtained your fluorescent protein in a pFA6a vector with a desired selectable marker (*kanMX6*, *hphMX6*, *natMX6*, and *bleMX6* etc.). These vectors are available via Addgene.
      **CRITICAL:** The primers need to be purified by PAGE (Polyacrylamide Gel Electrophoresis).
      ***Note:*** The restriction sites upstream of the encoding sequences for the epitope tag create a linker sequence RIPGLIN or RIPGLIK between the POI and the C-terminal tag. For certain proteins, longer flexible linkers containing Glycine or Serine may be desired to increase the functionality of the tagged proteins.^4^^,^^13^
      ***Note:*** Once the primers have arrived, spin down the primer dry powders in the tubes by using the “short” function on a microcentrifuge and resuspend them to a final concentration of 50 μM in sterile ddH_2_O, and then store them at −20°C.
2. Amplify linear DNA for transformation [∼3 hr].
  ***Note:*** For efficient gene targeting by homologous recombination in *S. pombe* cells, the genes for the epitope tag and associated selectable marker cassette need to be amplified by PCR to obtain highly concentrated DNA that contains the 70 nt of homology to the POI gene on both 5′ and 3′ ends.
  a. Set up the following PCR master mix on ice:

**Table:** 

Reagents (final concentration)	Volume (μL)	Stock
Sterile ddH_2_O	198	
Expand buffer 2 with MgCl_2_ (1×)	60	5× for HIFI+
dNTP mix (0.2 mM)	30	2 mM
Forward primer (0.5 μM)	3	50 μM
Reverse primer (0.5 μM)	3	50 μM
DNA template (∼3 ng/μL)	3	∼0.3 μg/μL
Expand HIFI polymerase (0.75 unit/20 μL)	2.25	5 units/μL
Total volume	300
    **CRITICAL:** We find that the Expand High-Fidelity PCR System has the best results in terms of fidelity, reliability, and DNA yields. Other PCR systems that we tried struggled to work with the long primers reliably or had unacceptable high mutation rates.
    ***Note:*** We split the 300 μL master mix equally into six or three PCR tubes. This allows the PCR reaction mix to change temperature more efficiently.
  b. Run the following PCR reaction.

**Table:** 

Steps	Temperature	Time	Cycles
Initial Denaturation	94°C	2 min	1
Denaturation	94°C	30 sec	20
Annealing	55°C^a^	30 sec	
Extension	68°C	1 min/kb	
Final extension	68°C	7 min	1
Hold	4°C	Forever	

a The annealing temperature is subject to change depending upon the melting temperatures of the primers used.
    ***Note:*** The annealing temperature is calculated based on the 20 nt at the 3′ end of homology to the plasmid DNA but not including the 70 nt on the 5′ end of each primer that adds homology to the gene of interest. See troubleshooting 1.
    ***Note:*** The number of cycles is set to 20 to obtain enough DNA needed for the transformation while minimizing the risk for point mutations to be introduced.
  c. Analyze the PCR product via gel electrophoresis.
    i. Prepare a 1% agarose gel with SYBR Safe DNA stain.
    ii. Load 10 μL of the PCR product with 1× loading dye into a 1% agarose gel alongside 10 μL (1 μg) of a 1 kb DNA Ladder in an adjacent well.
    iii. Run for ∼30 min at 100 V.
      ***Note:*** The time and voltage can be modified depending on the gel and gel tank being used.
    iv. Image the gel and confirm the presence of the DNA band at the correct size.
  d. Purify the PCR product using the Qiagen MinElute kit, eluting DNA with 10 μL EB buffer.
    ***Note:*** Highly concentrated DNA is required for efficient transformation. Thus, all the DNAs (∼4 μg) from the 300 μL reaction are loaded and purified using one column. It is important to add 10 μL of 3 M sodium acetate at pH 5.0 to the PCR product so that the DNAs can bind to the column efficiently.
    **Pause point:** The purified PCR DNA can be stored at −20°C (for ≥ 12 months), while you prepare the yeast culture.
3. Transform *S. pombe* cells.
  ***Note:*** This section is modified slightly from Bähler et al.^2^ The days for this transformation sub-protocol will be marked for clarity.
  a. Day 1: In the morning, inoculate 6–10 mL starter culture of wild type cells in the standard YE5S or YE5S-adenine (for diploid cells) liquid medium at 25–30°C.
    ***Note:*** We store our fission yeast strains at −80°C in medium with 25% glycerol and “wake” the strains up on YE5S or YE5S-adenine (for diploid cells) plates at 25 or 30°C. We grow cells for ≥36 hr (restreaking once or twice on the plate) before inoculating cells into liquid culture.
    ***Optional:*** We commonly transform wild-type cells (JW81 *h*^*-*^*ade6-M210* leu1-32 *ura4-D18* or JW729 *h*^*+*^*ade6-M210 leu1-32 ura4-D18*) to obtain strains expressing POI with different tags. Once confirmed, we cross the strains to a cell cycle marker or other strains that we are interested in.
  b. Day 1: In the evening, dilute the culture.
    i. Measure the Optical Density (OD) of the liquid culture at 595 nm.
    ii. Dilute the culture to OD_595_ = ∼0.02.
      **CRITICAL:** The culture must be diluted to ensure that the strain remains growing in the early exponential phase (OD_595_ ≤ 0.50).
      ***Note:*** Cells grow faster at a higher temperature, take this into account for your dilutions. We grow cells in liquid medium in baffled flasks (to facilitate air transfer) for 36 to 48 hr before transformation. The OD_595_ should be below 0.5 ideally in the morning of Day 2. The doubling time is about 3.5 hr at 25°C. If your strains are grown at 30°C then you may need to dilute to a lower OD_595_.
  c. Day 2: Dilute the culture in the morning.
    i. Measure the OD_595_ of the liquid culture.
    ii. Dilute the culture to 0.05 OD_595_.
  d. Day 2: Dilute the culture in the evening.
    i. Measure the OD_595_ of the liquid culture.
    ii. Dilute the culture to 0.02 OD_595_.
      ***Note:*** Increase the total volume of the culture to obtain enough cells for the transformation.
  e. Day 3: Measure the OD_595_ of the liquid culture.
    i. If the OD is above 0.5, then dilute to 0.25.
    ii. Wait for the culture to reach OD_595_ = 0.4 to 0.5.
  f. Turn on the 42°C water bath.
  g. Harvest 40–50 mL of cells by centrifuging in a 50 mL sterile tube for 5 min at 1,100 × *g* at a temperature between 22–24°C.
  h. Wash cells with a volume of sterile ddH_2_O equal to the volume of culture and repeat the centrifugation.
  i. Remove the supernatant and re-suspend the cell pellet in 1 mL of sterile ddH_2_O.
  j. Transfer the cell suspension into a sterile 1.5 mL microcentrifuge tubes (label the tubes in advance).
  k. Collect cells by centrifuging at 4,600 × *g* for 30 sec at 22–24°C in a microcentrifuge. Remove as much supernatant as possible, then resuspend the cell pellet in 1 mL 1× LiAc/TE.
  l. Collect cells by centrifuging at 4,600 × *g* for 30 sec at 22–24°C. Remove the supernatant, and then resuspend cells in 1× LiAc/TE to a final volume of 100 μL (cells plus liquid).
    **CRITICAL:** Since the final volume will be 100 μL, this means that you need only add ∼10 to 20 μL of 1× LiAc/TE at this step.
  m. Add 2 μL carrier DNA [10 mg/ mL ssDNA from herring testes (Clontech)] and 10 μL of the amplified and purified PCR DNA to the cells and vortex gently, and then incubate for 10 min at 22–24°C.
    ***Note:*** We find that stirring gently with the sterile pipet tip also achieves the mixing required without damaging the cells.
    ***Note:*** All vortexing was done on a Fisher Vortex Genie 2, but a similar tabletop vortex will work sufficiently.
  n. Add 260 μL sterile 40% PEG3350/LiAc/TE; mix by vortexing or by pipetting up and down gently.
    ***Note:*** The PEG causes the solution to be very sticky, so all pipetting at this step must be done slowly.
  o. Incubate for 60 min at 22–24°C. Mix 2 or 3 times during the incubation.
    ***Note:*** Because the cells may precipitate, it is best to lay the tube on its side.
  p. Add 43 μL DMSO, mix by vortexing or by pipetting up and down gently, then heat shock at 42°C for 5 min.
    **CRITICAL:** Do not allow the heat shock to extend longer than 5 min. The cells are fragile and may die from prolonged exposure to 42°C.
  q. Collect cells by centrifuging at 4,600 × *g* for 30 sec at 22–24°C. Remove the supernatant.
  r. Wash cells once by adding 1 mL sterile ddH_2_O, pipetting gently up and down to completely resuspend the cells.
  s. Collect cells by centrifuging at 4,600 × *g* for 30 sec at 22–24°C. Remove the supernatant, and re-suspend cells in 500 μL sterile ddH_2_O.
  t. Plate the cells evenly onto 2 plates using 3–5 sterile glass beads by shaking gently.
    ***Note:*** At this step, we use YE5S plates for transforming DNAs containing the *kanMX6*, *hphMX6*, or *natMX6* antibiotic selectable marker. We use YE5S - adenine plates for diploid strains. We use EMM5S - uracil selection plate directly for the *ura4*^*+*^ selectable marker.
  u. Once the cells are spread evenly and the plates are not visibly wet, dump the glass beads, invert the plate, and incubate the plates at 25 or 30°C.
  v. Days 4 or 5: ∼24 hr (30°C) to 36 hr (25°C) after plating the cells, replica plate the cells onto selection plates: YE5S (haploids) or YE5S-ade (diploids) containing 100 mg/L G418 (Geneticin), 150 mg/L Hygromycin B, or 50 mg/L ClonNAT (Nourseothricin Sulfate), dependent on the selectable marker.
    ***Note:*** The incubation temperature depends on the predicted temperature-sensitivity of constructed strains. Because temperature-sensitive mutants are more common than cold-sensitive mutants, it is safer to grow cells at 25°C, or one plate at 25°C and the other at 30°C.
    ***Note:*** Replica plating is the process of copying cells between plates using a sterile velvet. In this case, we copy cells from the non-selecting medium to the medium with appropriate antibiotics.
    ***Note:*** G418 selection has more tiny background colonies (which are not real transformants) than hygromycin or ClonNAT selection.
  w. Incubate the plates at 25 or 30°C in a plastic bag (or sealed with parafilm).
    ***Note:*** The colonies will take four to six days to grow up dependent on the temperature. Normally, tens to hundreds of colonies can be expected. See troubleshooting 2.
    ***Note:*** If the strongest *nmt1* promoter is used for tagging at the N-terminus, consider using plates with 5 mg/L thiamine to avoid overexpression effects.
  x. After 4–6 days, when the colonies are big enough to restreak, pick up the colonies using a sterile applicator stick and restreak them onto new selection plates (with antibiotics or without uracil).
  y. After ∼12 hr, the cells are ready to be restreaked, analyzed (genomic DNA isolation and PCR checking), or visually screened (by phenotype or fluorescence signals).
  z. Mark the positive or potentially positive transformants for further analysis or storage.
    **Pause point:** The strains can be stored at −80°C in YE5S + 25% Glycerol and await further analysis.

### Confirm the transformants by PCR

**Timing: 1 day**

4. Extract the Genomic DNA of the fission yeast transformants.

***Note:*** We find that the Genomic DNA Extraction protocol from Lõoke et al., 2011 to be a reliable way to obtain the genomic DNA with sufficient quality and quantity from *S. pombe*.^20^ However, other protocols can also be used. The goal is to have the genomic DNA good enough for PCR analysis.

**Pause point:** Once the genomic DNA is extracted, it can be stored at −20°C for ≥ 12 months.

**Figure 1:**
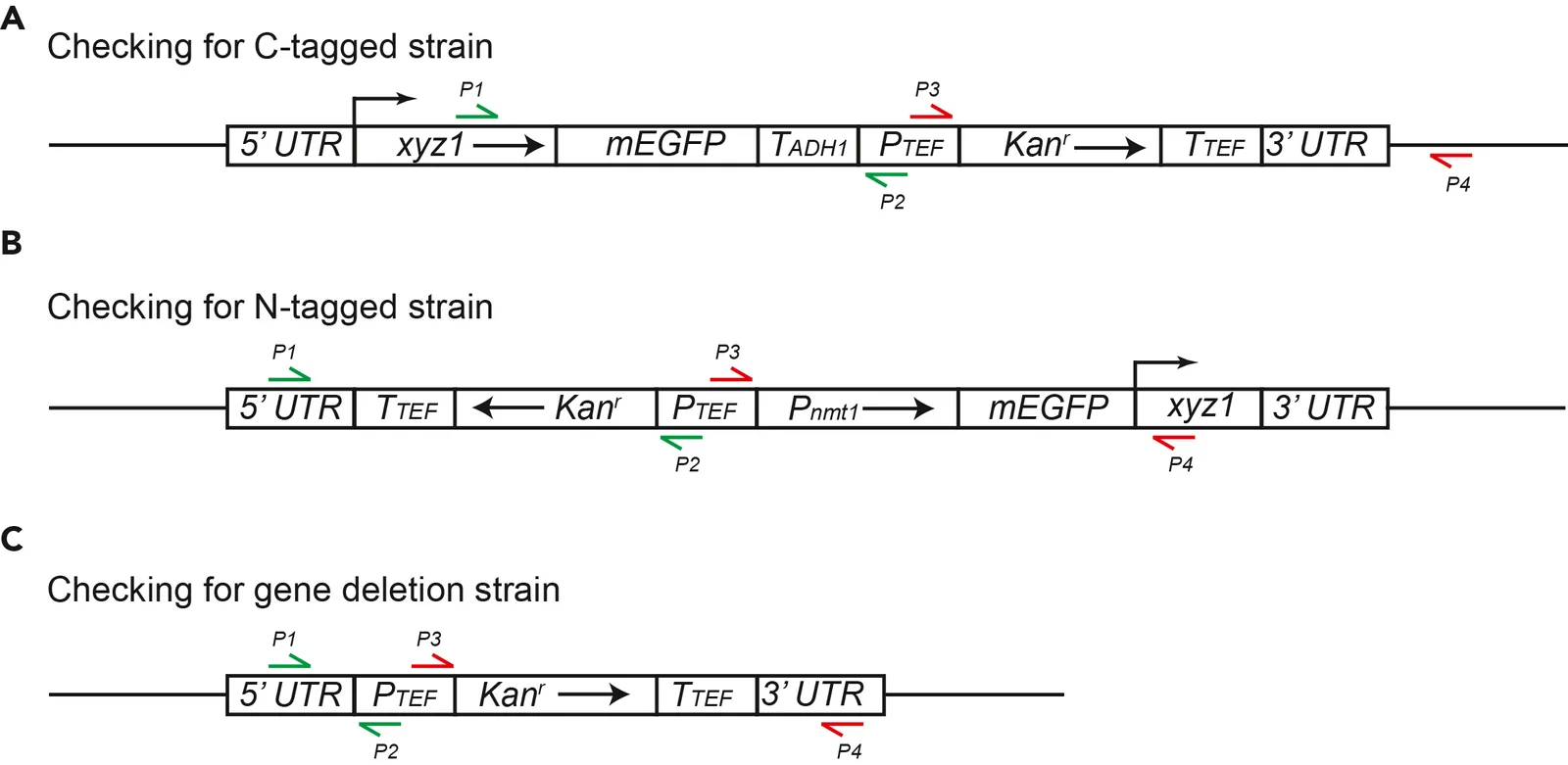
PCR-based strategy to verify correct integration of gene-targeting cassettes by checking both junctions of the DNA insertion (A) Primer design for C-terminal tagging of the gene *xyz1.* Checking primers (20–25 nt) are designed in pairs (the paired forward and reverse primers are shown in the same color). For the 5′ junction, the forward primer binds within the *xyz1* ORF and the reverse primer within the *TEF* promoter for the antibiotic resistance marker (e.g., *kan*^*r*^). For the 3′ junction, the forward primer binds the *TEF* promoter and the reverse primer binds ∼200–400 nt downstream of the STOP codon of the gene *xyz1*. (B) Primer design for N-terminal tagging of the gene *xyz1.* For the 5′ junction, the forward primer binds to the 5′ UTR and the reverse primer in the *TEF* promoter of the antibiotic resistance gene (*kan*^*r*^). For the 3′ junction, the forward primer binds the *TEF* promoter or the *nmt1* promoter and the reverse primer binds the gene *xyz1* (∼200–400 nt downstream of the START codon). These primer pairs can also be used when the native promoter is used instead of the *nmt1* promoter. (C) Primer design for gene deletion strains. For the 5′ junction, the forward primer binds to the 5′ UTR of gene *xyz1* and the reverse primer in the *TEF* promoter of the antibiotic resistance gene (*kan*^*r*^). For the 3′ junction, the forward primer binds to the *TEF* promoter and the reverse primer binds ∼200–400 nt downstream of the STOP codon of the deleted gene. P1 and P4 can also be used to amplify the whole cassette inserted if the length of the *xyz1* gene is different from the deletion cassette.
6. Set up two PCR reactions using each pair of primers to amplify the junctions of the insertion for each new strain. Run the PCR based on the preferred polymerase system of choice.

***Note:*** The type of polymerase used here is not critical unless the PCR products will be sequenced. We normally use the robust and cost-effective Taq polymerase.

5. Four 20–25 nt primers are needed to check both junctions of DNA insertion site into the genome during gene targeting (See Figure 1). Using C-terminal tagging as an example, these four primers are needed:
  a. Forward primer P1: binds to the upstream of the stop codon in the POI gene to check the 5′ insertion site.
    ***Note:*** Ideally, the designed primer should have a similar annealing temperature to its complement below and be only 200–400 nt upstream from the stop codon.
  b. Reverse primer P2: binds to the promoter *P*_*TEF*_ of the transforming DNA to check the 5′ insertion site.
    ***Note:*** We use the following primer: 5′-GTATGGGCTAAATGTACGGGCGACAGTCAC-3’. But other primers that bind in the cassette may work as well.
  c. Forward primer P3: binds to the promoter *P*_*TEF*_ of the transforming DNA (no overlapping with the Reverse primer 1) to check the 3′ insertion site.
    ***Note:*** We use the following primer: 5′- GCTAGGATACAGTTCTCACATCACATCCG-3’. But other primers that bind in the cassette may work as well.
  d. Reverse primer P4: binds to 3′ UTR or its downstream sequences for the POI gene to check the 3′ insertion site.
    ***Note:*** Ideally, the designed primer should have a similar annealing temperature to its complement above and be only 200–400 nt downstream from the stop codon.
    ***Note:*** Ideally, gene-specific primers (Forward primer P1 and Reverse primer P4) bind to the POI gene at a few hundred base pairs away from the insert site so that the DNA bands can be easily resolved on agarose gel.

7. Analyze the PCR products via agarose gel electrophoresis as done in step 2c.

### Strain storage

**Timing: 1 or 2 days**

8. Restreak positive or potentially positive colonies (ideally ≥3) onto a new selection plate (cover at least a quarter of the petri dish) and grow for one (30°C) or two days (25°C).
9. Name or number the new strain in the lab master strain list so that it can be easily recalled for future usage.
10. Add 1 mL of sterile YE5S liquid media with 25% glycerol to a sterile screw-capped cryotube. Write down the strain name and main genotype on the side of the tube.
11. Use a sterile wood applicator stick to transfer as many cells as possible to the cryotube. One stick per colony.
12. Mix thoroughly, tighten the cap, and put a round sticker with the strain number on the top of the cap.
13. Store the tube at −80°C.

**Pause point:** The strain can remain at −80°C for ≥ 10 years. However, for strains that are “woken up” from −80°C, they need to grow ∼36 hr on a YE5S agar plate before experiments.

### Confirm the functionality of the tagged POI

**Timing: 2 days or ∼10 days if genetic interactions are needed**

14. Using the fresh cells from above and wild type cells, restreak the cells onto YE5S + Phloxin B and YE5S plates (and other media if necessary) and incubate the plates at various temperatures, such as 20, 25, 30, 32, and 36°C.
15. After 12 to 24 hr, swab cells from the YE5S plates onto a microscope slide with 1 to 2 μL of media using a sterile toothpick, then cover the cells with a coverslip.
16. Observe and/or image the cells on a microscope to assess cell morphology and protein localization.
17. Scan the PB plates several times from 12 to 48 hr after restreaking the cells to check the cell color.

***Note:*** Phloxin B is a red colored live dye that can enter *S. pombe* cells. Healthy cells can pump it out and so remain white and slightly pink. By contrast, the sick and dying cells cannot pump out the dye efficiently, which causes the cells to appear red. The redder the color, the unhealthier the cells are.

18. Assess your strains by analyzing the growth rate, cell morphology, and protein localization in liquid culture at early exponential phase.

***Note:*** It is possible that epitope tags subtly affect the localization and function of the tagged protein even expressed under the control of its native promoter.^13^ Therefore, it is necessary to implement more rigorous and sensitive assessments than simply analyzing cell phenotype and protein localization, like testing the functionality using genetic interactions. For example, if a mutation in the POI is known to have a strong synthetic interaction with “mutation A”, the synthetic interaction between the tagged POI and mutation A can be tested. If the double mutant resembles mutation A, the tagged POI is functional. If the double is more defective than mutation A, then the tagged POI is not fully functional.

## Key resources table

**Table:** 

REAGENT or RESOURCE	SOURCE	IDENTIFIER
**Antibodies**		

Rabbit anti-GFP	Novus Biologicals	Cat# NB600-308; RRID:AB_10003058
α-GFP/YFP monoclonal	Roche	Cat# 11814460001; RRID:AB_390913
α-Myc	Santa Cruz Biotechnology	Cat# 9E10; RRID:AB_2266850
α-mNeonGreen	Proteintech	Cat# 32F6; RRID:AB_2827566
α-tubulin	Woods et al.^21^	TAT1
α-mouse IgG	Sigma	Cat# A4416; RRID:AB_258167

**Chemicals, peptides, and recombinant proteins**		

Adenine	Sigma	A9126
Agar	Difco	214530
Amylose beads	New England Biolabs	E8021S
BioMax MR film	Kodak	Z350370
Biotin	Sigma	B4501
Boric acid	Sigma	B9645
CaCl_2_ · 2H_2_O	Fluka	21097
Citric acid	Sigma	C2404
ClonNat (Nourseothricin sulfate)	Gold Bio	N-500–100
CuSO_4_ · 5H_2_O	Sigma-Aldrich	209198
Dextrose	Sigma-Aldrich	G8270
dNTP	New England Biolabs	N0447L
Dynabeads	Invitrogen	10004D
EDTA	Sigma	E6758
FeCl_3_ · 6H_2_O	Fluka	44944
G418 (Geneticin)	Gibco	11811–031
Gelatin	Sigma-Aldrich	G2500
Glacial acetic acid	Fisher	A38S-212
Glycerophosphate	EMD Millipore	35675
HCl	Fisher	A144-212
HEPES	Sigma-Aldrich	H4034
Histidine	Sigma	H-8000
Hygromycin B	Gold Bio	H-270–1
Inositol	Sigma	I7508
KCl	Sigma	P9541
KH_2_PO_4_	Sigma-Aldrich	P9791
KI	Sigma-Aldrich	P8166
Lamelli sample buffer	Bio-RAD	1610747
Leucine	Sigma	L-8000
Lithium acetate · 2H_2_O	Sigma-Aldrich	L6883
Lysine	Sigma	L5626
Methanol	Fisher	A412SK-4
MgCl_2_ · 6H_2_O	Sigma-Aldrich	M2393
MnSO_4_ · H_2_O	Sigma-Aldrich	M7634
Sodium molybdate (Na_2_MoO_4_ · 2H_2_O)	Sigma	M1651
Na_2_HPO_4_	Sigma-Aldrich	S5136
Na_2_SO_4_	Sigma-Aldrich	S5640
Na_3_VO_4_	Sigma-Aldrich	450243
NaCl	Fisher	BP358-10
NaF	Sigma-Aldrich	S1504
NH_4_Cl	Sigma-Aldrich	213330
Nicotinic acid	Sigma	N0761
NP-40	Sigma	I3021
*n*-Propyl-gallate (PG)	Sigma	P3130
Pantothenic acid	Sigma-Aldrich	P6292
PEG 3350	Sigma	P4338
Phloxin B	Sigma	P4030
PMSF	Sigma-Aldrich	P7626
Potassium phthalate monobasic	Sigma-Aldrich	179922
Precision plus protein ladder	Bio-RAD	161–0374
Protease inhibitor cocktail cOmplete	Roche	11873580001
SuperSignal west maximum sensitivity substrate	Thermo Fisher Scientific	34096
SYBR Safe DNA Gel Stain	Invitrogen	S33102
Talon metal affinity resin	TAKARA	635502
Tris base	Sigma-Aldrich	T6066
Tween 20	Sigma	P7949
Uracil	Sigma	U0750
Yeast extract	Bacto	212750
ZnSO_4_ · 7H_2_O	Sigma	Z0251

**Experimental models: Organisms/strains**		

*h*^*-*^*ade6-M210* leu1-32 *ura4-D18*	Wu lab^1^	JW81
*h*^*+*^*ade6-M210 leu1-32 ura4-D18*	Wu lab^1^	JW729
*h^-^ pmo25-mECitrine:kanMX6 ade6-M210 leu1-32 ura4-D18*	Wu lab^1^	JW7943
*h*^*-*^*pmo25-tdTomato:natMX6 ade6-M210 leu1-32 ura4-D18*	Wu lab^1^	JW7944

**Oligonucleotides**		

Pmo25 C-tag F	Forward primer for Pmo25 C terminal tagging	5'-TACTGACCGGAAAAATGATGAGCAGTTTAATG ATGAACGCGCATTTGTAATAAAACAGATTGA ACGCTTACGGATCCCCGGGTTAATTAA-3'
Pmo25 C-tag R	Reverse primer for Pmo25 C terminal tagging	5'-TCCTTTGTAAATATAGGATGGACAACAGCATGAT TTTGAAAGACATGAGCACATTGGTGAGTAACA AGAGGAATTCGAGCTCGTTTAAAC-3'
Pmo25 C-tag chk F	Forward checking primer	5'-GTTGCTAATCCTGAAAAGTCGG-3'
Pmo25 C-tag chk R	Reverse checking primer	5'-GAAGAAAGAAAAGGCTGTGGAA-3'

**Recombinant DNA**		

pFA6a-kanMX6-P3nmt1-mEGFP	Wu lab^1^	JQW72
pFA6a-kanMX6-P41nmt1-mEGFP	Wu lab^1^	JQW75
pFA6a-kanMX6-P81nmt1-mEGFP	Wu lab^1^	JQW78
pFA6a-mEGFP-kanMX6	Wu lab^1^	JQW85
pFA6a-mYFP-kanMX6	Wu lab^1^	JQW86
pFA6a-13Myc-hphMX6	Toda Lab.^22^	JQW121
pFA6a-tdTomato-natMX6	Sawin Lab^23^	JQW133
pFA6a-mECitrine-kanMX6	Wu lab^1^	JQW228
pFA6a-mYFP-hphMX6	Wu lab^1^	JQW346
pFA6a-mCherry-hphMX6	Wu lab^1^	JQW347

**Software and algorithms**		

Fiji Software	https://fiji.sc (Schindelin et al.)^24^	RRID:SCR_002285
Volocity	PerkinElmer	N/A
NISElements	Nikon	N/A

**Other**		

Fisher vortex genie 2	Fisher Scientific	12–812
PES membrane 0.22 μm filter unit	Millipore-Sigma	SLGPR33RS
iBright CL1500 imager	Thermo Fisher Scientific	RRID:SCR_026565
Lyophilizer	Aapptec	7001100115
SoRa Spinning Disk Confocal Microscope Ti2	Nikon	Yokogawa CSU-W1
100× Plan Apo λD objective, N.A. 1.45	Nikon	MRD71970
Microscope slides (3″ × 1″ × 1mm)	VWR	16004–430
Cover glass (22mm × 22mm), No. 1.5	Corning	2850–22
Expand high fidelityPLUS PCR system	Sigma-Aldrich	03300226001
PCR machine	Bio-RAD	T100
MinElute PCR purification kit for PCR cleanup	Qiagen	28004
Loading tips for SDS PAGE	USA Scientific	1022–0600
SDS PAGE gels	Bio-RAD	4561093
Mini-sub cell GT horizontal electrophoresis system	Bio-RAD	1704467
1 kb DNA ladder	New England Biolabs	N3232L
10× orange loading dye	Sigma-Aldrich	O3756
Carrier DNA from herring testes	Clontech/Takara	630440
BSA	Sigma	A6003
Non-fat dry milk	Kroger	Kroger brand
PVDF transfer membranes, 0.45 μm Immobilon-P	Sigma Millipore	IPVH 00010
Whatman 3MM CHR	Cytiva	3030–861
1.5 mL low adhesion centrifuge tube	USA Scientific	1415–2600
Nalgene cryogenic vial	Thermo Scientific	5000–0020

## Materials and equipment

**Table:** YE5S

Reagent	Final concentration	Amount
ddH_2_O	N/A	980 mL
Yeast Extract	5 g/L	5 g
Dextrose	167 mM	30 g
Agar	18 g/L	18 g
Uracil	2.01 mM	0.225 g
Adenine	0.61 mM	0.225 g
Leucine	1.72 mM	0.225 g
Histidine	1.45 mM	0.225 g
Lysine	1.23 mM	0.225 g
Total	–	1 L

• Once the mixture is prepared and dissolved, autoclave the liquid for 20 min using the liquid cycle with a magnetic stir bar. Stir the medium to mix and cool on a stir plate.
• To make agar plates, pour ∼25 mL of the media into a petri dish once the solution cools to ∼55°C.
• After 2–3 days, store the plates in a plastic bag at 4°C.

**YE5S liquid:** Do not add agar, and autoclave in two 500 mL bottles.

**YE5S + Phloxin B:** Add 1 mL of 1,000× PB (5 g/L) stock solution (at 4°C) per liter after the solution cools to ∼55°C after autoclaving. Store the plates wrapped in aluminum foil to protect the Phloxin B from light.

**YE5S + G418 (Geneticin)**: Add 100 mg of G418 powder per liter or 1 mL 1000× stock solution (100 mg/mL, stored at −20°C) after the medium cools to ∼55°C after autoclaving.

**YE5S + ClonNAT (Nourseothricin Sulfate)**: Add 1 mL of 1000× stock solution clonNAT (50 mg/mL, stored at −20°C) per liter after the medium cools to ∼55°C after autoclaving.

**YE5S + Hygromycin**: Add 3 mL of Hygromycin B (50 mg/mL in ddH_2_O, filtered and stored at 4°C) per liter after the medium cools to ∼55°C after autoclaving.

***Note:*** Plates can be stored at 4°C for ≥ 24 months. Liquid media can be stored at 22–24°C for ≥ 24 months.

**Table:** 50× Salts

Reagent	Final concentration	Amount for 1 L
MgCl_2_ · 6H_2_O	0.26 M	52.5 g
CaCl_2_ · 2H_2_O	4.99 mM	0.735 g
KCl	0.67 M	50 g
Na_2_SO_4_	14.1 mM	2 g

Store at 4°C for ≥ 24 months.

**Table:** 1,000× Vitamins

Reagent	Final concentration	Amount for 1 L
Pantothenic acid	4.20 mM	1 g
Nicotinic acid	81.2 mM	10 g
Inositol	55.5 mM	10 g
Biotin	40.9 μM	10 mg

Store at 4°C for ≥ 24 months, or −20°C for ≥ 10 years.

**Table:** 10,000× Minerals

Reagent	Final concentration	Amount for 1 L
H_3_BO_3_ (Boric acid)	80.9 mM	5 g
MnSO_4_	23.7 mM	4 g
ZnSO_4_ · 7H_2_O	13.9 mM	4 g
FeCl_3_ · 6H_2_O	7.40 mM	2 g
Molybdic acid	2.47 mM	0.4 g
KI	6.02 mM	1 g
CuSO_4_ · 5H_2_O	1.60 mM	0.4 g
Citric acid	47.6 mM	10 g

Store at 4°C for ≥ 24 months, or −20°C for ≥ 10 years.

**Table:** EMM5S- Component 1

Reagent	Final concentration	Amount
ddH_2_O	N/A	870 mL
Potassium phthalate monobasic	14.7 mM	3 g
Na_2_HPO_4_ anhydrous	15.5 mM	2.2 g
NH_4_Cl	93.5 mM	5 g
50× salts stock (at 4°C)	1×	20 mL
10,000× minerals stock (at 4°C)	1×	100 μL
Agar	20 g/L	20 g
Uracil	2.01 mM	0.225 g
Adenine	0.61 mM	0.225 g
Leucine	1.72 mM	0.225 g
Histidine	1.45 mM	0.225 g
Lysine	1.23 mM	0.225 g
Total	–	900 mL

**Table:** EMM5S- Component 2

Reagent	Final concentration	Amount
ddH_2_O	N/A	87.5 mL
Dextrose	111 mM	20 g
Total	–	100 mL

• Once the mixture is prepared and dissolved, autoclave the liquid for 20 min using the liquid cycle. Stir the medium to mix and cool on a stir plate.
• Add component B to component A when the medium cools to ∼70°C.
• Add 1 mL of 1,000× vitamins stock after the medium cools to ∼55°C.
• For making plates, pour ∼25 mL of the medium once it cools to ∼55°C into a petri dish.
• After 2–3 days, store the plates in a plastic bag at 4°C.

**EMM5S + Thiamine:** Add 1 mL 1,000× stock of thiamine (5 mg/mL at 4°C) after autoclaving and the media cools to ∼55°C.

***Note:*** EMM5S plates can be stored at 4°C for ≥ 24 months. Liquid media can be stored at 22–24°C for ≥ 24 months.

***Note:*** The above recipes were based on the work of J. Petersen and P. Russell.^25^

**Table:** 10× TE

Reagent	Final concentration	Amount
ddH_2_O	N/A	∼80 mL
Tris-HCl (1 M stock, pH 7.5)	100 mM	10 mL
EDTA (0.5 M stock, pH 8.0)	10 mM	2 mL
HCl	N/A	To pH 7.5 (∼0.2 mL)
Total	–	100 mL

Store at 22–24°C for ≥ 24 months.

***Note:*** After pH is 7.5, add ddH_2_O to 100 mL. Autoclave.

### 10× LiAc

To 1 M Lithium Acetate, add 1:10 diluted glacial acetic acid to pH 7.5. Usually ∼550 μL per 1 L. Filter to sterilize. Store at 22–24°C for ≥ 24 months.

**Table:** 1× LiAc/1× TE

Reagent	Final concentration	Amount
ddH_2_O	N/A	40 mL
LiAc (10×)	1×	5 mL
TE (10×)	1×	5 mL
**Total**	–	**50 mL**

Store at 22–24°C for ≥ 6 months.

**Table:** PEG/LiAc/TE

Reagent	Final concentration	Amount
ddH_2_O	N/A	9.75 mL
PEG 3350	40% (w/v)	8 g PEG
LiAC (10×)	1×	2 mL
TE (10×)	1×	2 mL
**Total**	–	**20 mL**

Filter to sterilize using a 0.22 μm filter.

Store at 22–24°C for ≥ 1 month or at 4°C for several months. Fresh solutions have a higher transformation efficiency.

**Table:** IP Buffer (for Pmo25)

Reagent	Final concentration	Amount
4-(2-hydroxyethyl)-1-piperazineethanesulfonic acid [HEPES], pH 7.5	50 mM	1.19 g
NaCl (5 M stock)	150 mM	3 mL
EDTA (0.5 M stock)	1 mM	0.2 mL
NP-40 (10% stock)	1%	10 mL
NaF	50 mM	0.21 g
Glycerophosphate (10× stock)	20 mM	10 mL
Na_3_VO_4_ (1000× stock)	0.1 mM	100 μL
PMSF (0.1 M stock)	1 mM	1 mL
EDTA free protease inhibitor tablets	N/A	2 tablets
ddH_2_O	N/A	Up to 100 mL
**Total**	–	**100 mL**

Store the IP buffer (without PMSF and EDTA free protease inhibitor tablet) at 4°C for up to 12 months.

***Note:*** The PMSF is added just before use.

*Alternatives***:** Triton and CHAPS can be added to replace NP-40. Both added to a final concentration of 0.5%.

**Table:** Phosphate-Buffered Saline (PBS) Buffer

Reagent	Final concentration	Amount
NaCl	137 mM	8 g
KCl	2.7 mM	0.2 g
Na_2_HPO_4_	10 mM	1.44 g
KH_2_PO_4_	2 mM	0.24 g
HCl	N/A	Add to adjust pH to 7.4
ddH_2_O	N/A	Up to 1 L
**Total**	–	1 **L**

Store at 4°C for ≥ 12 months.

**Table:** Wash Buffer I

Reagent	Final concentration	Amount
4-(2-hydroxyethyl)-1-piperazineethanesulfonic acid [HEPES], pH 7.5	50 mM	1.19 g
NaCl (5 M stock)	150 mM	3 mL
EDTA (0.5 M stock)	1 mM	0.2 mL
NP-40 (10% Stock)	0.1%	1 mL
ddH_2_O	N/A	Up to 100 mL
**Total**	–	**100 mL**

Store at 4°C for ≥ 12 months**.**

**Table:** Wash Buffer II

Reagent	Final concentration	Amount
4-(2-hydroxyethyl)-1-piperazineethanesulfonic acid [HEPES], pH 7.5	50 mM	1.19 g
NaCl (5 M stock)	150 mM	3 mL
EDTA (0.5 M stock)	1 mM	0.2 mL
ddH_2_O	N/A	Up to 100 mL
**Total**	–	**100 mL**

Store at 4°C for ≥ 12 months.

**Table:** 1× TBST

Reagent	Final concentration	Amount
ddH_2_O	N/A	949 mL
Tris-HCl pH 7.5 (1 M)	0.02 M	20 mL
NaCl (5 M)	0.15 M	30 mL
Tween 20 (100% w/v)	0.1%	1 mL
**Total**	–	**1 L**

Store at 22–24°C for ≥ 12 months.

**Table:** 50× TAE

Reagent	Final concentration	Amount
ddH_2_O	N/A	Up to 1 L
Tris Base	2.0 M	242 g
Glacial Acetic acid	1.0 M	57.2 mL
EDTA (0.5 M stock)	0.05 M	100 mL
**Total**	–	**1 L**

Store at 22–24°C for ≥ 12 months.

**Table:** 1× TAE

Reagent	Final concentration	Amount
50× TAE	1×	20 mL
ddH_2_O	N/A	980 mL
**Total**	–	**1 L**

Store at 22–24°C for ≥ 12 months.

### 1% agarose gel

Add 0.5 g of agarose to 50 mL of 1× TAE. Melt the agarose in a microwave for ∼90 seconds, stirring occasionally. Allow to cool to where you can touch it before adding the DNA stain. Store at 4°C ≥ 1 day.

## Step-by-step method details

### Prepare the yeast strains for microscopy

**Timing: 2.5 days**

The *S. pombe* strain with the tagged POI must be grown exponentially in liquid cultures before imaging and quantitative analysis. This section of the protocol is detailed in our previous publication.^17^

1. Grow the cells in liquid culture.
  a. Approximately 36 hr before imaging, using fresh cells on a plate and a sterile wooden applicator, inoculate the strain with the tagged POI into 10 mL of YE5S liquid media in a baffled sterile flask and incubate in a water bath at 25°C, shaking at 150 rpm.
    ***Note:*** Baffled flasks are better for growing yeasts since they facilitate oxygen diffusion while shaking.
    ***Note:*** The cells must grow for ∼36 hr to ensure they are in the exponential phase of growth for imaging.
  b. While the strain is growing, every 8–16 hr check the OD_595_ of the culture and dilute it to an OD_595_ between 0.02–0.05.
    ***Note:*** If the culture is growing at a higher temperature, it needs to be diluted to a lower OD_595_ or to have the timing between dilutions changed to ensure the cells do not overgrow (OD_595_ > 0.5).
    ***Note:*** For microscopy (the next step), only a few mL of culture is needed. However, for Western blot and other purification procedures, the liquid culture will need to be scaled up to ∼500 mL by the evening dilution.
  c. On the day the cells will be experimented on, check the OD_595_ of the culture and dilute (if needed). If this is for imaging, then dilute it to ensure that the OD_595_ is between 0.2 and 0.5 when imaging begins.
2. Prepare microscope slides.
  a. Add 0.25 g of gelatin to 0.9 mL of YE5S or EMM5S liquid medium in a microcentrifuge tube, mix immediately by shaking vigorously to mix the gelatin.
    ***Note:*** The medium to use depends on the experiments and/or strains. Cells grow faster and healthier in YE5S, but EMM5S has lower autofluorescence.
  b. Melt the gelatin on a hot plate at 65°C.
  c. Once melted, add 0.1 mL of n-propyl gallate (PG) to a final concentration of 5 μM (10× stock of 50 μM in YE5S or EMM5S kept at −20°C). Mix by inverting and then incubate in the hot plate.
    ***Note:*** PG helps quench free radicals that are generated during imaging.
  d. Wait for ∼15 min until the air bubbles disappear, then add 0.1 mL of the gelatin medium onto a microscope slide.
    ***Note:*** We recommend using a P1000 pipet tip to add 0.1 mL because the gelatin is highly viscous and may get stuck in a P200 tip.
    **CRITICAL:** Carry out this step swiftly, as the gelatin will harden within the pipet tip as you expel it. However, be careful not to introduce air bubbles onto the slide as these will complicate imaging.
  e. After ∼10 sec, spread the gelatin by pressing another microscope slide on top and clamp the ends of the slides with small paper binders.
    **CRITICAL:** Allow the gelatin medium on the slides to harden in the dark because PG is light sensitive. If the slides will not be used until >1 hr later, store them in a moist chamber in the dark.
  f. When it is time to use the slide, open and remove one slide from the gelatin pad. If the gelatin tears or crumples, use a different slide.
    *Alternatives:* To image cells using a cover glass bottom dish, please refer to our previous publication.^17^
3. Prepare the cells for microscopy.
  a. Transfer 0.9 mL of cell culture in YE5S from the flask to a microcentrifuge tube.
  b. Add 0.1 mL of PG from 10× stock in YE5S, mix, and incubate for ∼3 min in the dark.
    ***Note:*** If the cells will be imaged on an EMM5S + gelatin slide, wash the cells at least once with 1 mL EMM5S liquid medium, resuspend the cells in 0.9 mL EMM5S, and add 0.1 mL of PG from 10× stock in EMM5S, and incubate for ∼3 min in the dark.
  c. Spin down the cells at 845 × *g* for 30 sec.
  d. Remove the supernatant, leaving ∼15 to 20 μL medium to resuspend the cell pellet.
  e. Add ∼5 μL to the gelatin pad.
  f. Cover the cells with a 22 × 22 mm coverslip (No. 1.5), tap gently with the blunt end of 1 mL pipet tip to spread the cells, and seal the coverslip with VALAP (1:1:1 Vaseline: lanoline: paraffin) to minimize drying up.
    ***Note:*** One can pause briefly at this point, but the cells should not be left on the gelatin slide for hours, for they might overgrow and become too saturated, obscuring imaging and depleting nutrients from the gelatin.

### Image acquisition

**Timing: ∼30 min per strain for single image stacks, >1 h for most time-lapse movies**

With the strains prepared on a microscope slide with gelatin or an imaging dish, it is now time to obtain high resolution images of the POI.

**Figure 2:**
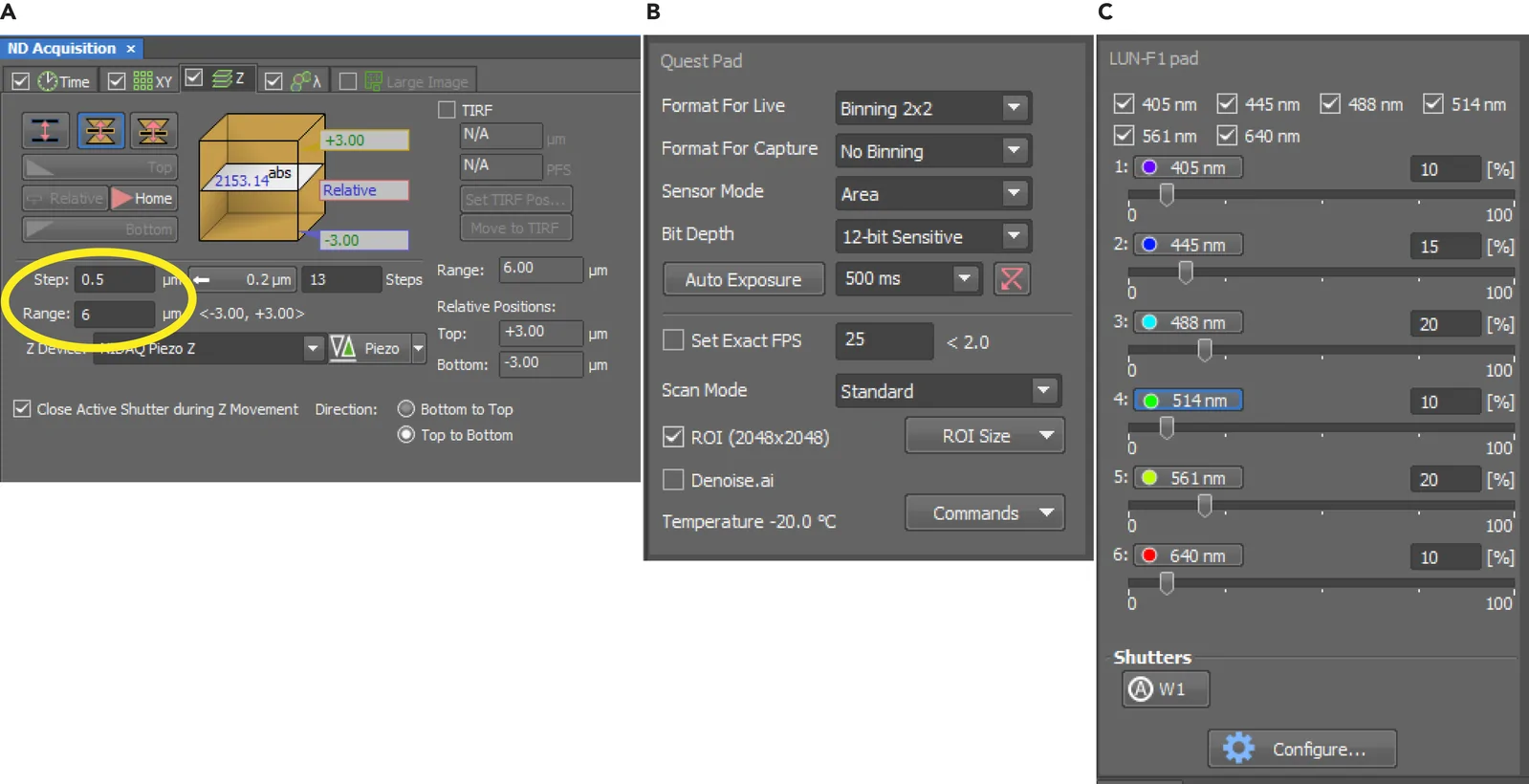
Nikon NIS-Elements acquisition screen showing key parameters for imaging Pmo25-mECitrine (JW7943) (A) The imaging panel for Z-slices. The “Step” and “Range” (circled) are set based on the experimental purpose and needed Z resolution. (B) The imaging settings. Optimizing the binning, exposure time, and ROI size are all necessary for obtaining quality images. (C) The laser power must be fine-tuned to minimize photobleaching and produce a clear image with enough signal to noise ratio for analysis.

4. Using Nikon NIS-Elements AR (Advanced Research), set up image acquisition (Figure 2).
  ***Note:*** We utilize a Spinning Disk Confocal System, which allows high temporal and spatial resolution live-cell imaging with manageable photobleaching and phototoxicity. Our previous publications provide details for this step.^1^^,^^13^^,^^14^^,^^16^ Though other microscopy systems are also mostly sufficient.
  ***Note:*** The exposure time and laser power used for imaging the POI depend on the fluorescent tag, the expression of the POI, and the other settings (numbers of images and time intervals).
  ***Note:*** The binning option depends on the camera and the trade-off between spatial resolution and signal intensity. Binning increases signal intensity but reduces spatial resolution. Because of the bigger pixel size, no binning is preferred for EMCCD camera. Binning (2 × 2) can be used for other cameras such as the CMOS due to its small pixel size.
  a. Set up the “Z” slices to obtain images across the Z-axis (Figure 2A).
    ***Note:*** We utilized varied spacing dependent on the experimental purpose in Ye, et al. 2025.^1^ For 3D reconstruction, the Nyquist-Shannon sampling theorem is needed.^26^
    ***Optional:*** For a time-lapse movie, lower laser power and shorter exposure time are used compared to single Z stacks. It is ideal to use multiple “XY” positions to obtain several movies at once if the time interval is long enough. However, as seen in Figure 5 in Ye, et al. 2025, a time lapse movie is not always necessary to obtain protein dynamics at the division site. Due to ring constriction and other trackable events, the protein kinetics at the division site can be obtained through imaging many asynchronous cells.^1^
  b. Add a drop of immersion oil onto the cover slip or the objective and load the sample onto the slide holder or dish holder.
  c. In brightfield or Differential Interference Contrast (DIC) mode, locate cells and focus at the middle focal plane.
    ***Note:*** We prefer DIC for better contrast and clearer cell morphology.
    **CRITICAL:** Do NOT locate or focus the cells for imaging using fluorescence channels. The lasers will bleach the signal and affect quantification. When changing the fluorescence channel settings, please ensure that the microscope setting is “Paused”, NOT “Live”. Only use “Live” when in DIC or brightfield mode.
  d. Confirm the imaging settings used (Figure 2).
    i. This can be accomplished by taking test images using the complete acquisition sequence (neglecting multiple XY points).
    ii. This can also be accomplished by simply clicking the “Capture” button when paused on the focused cells. This will obtain an image at the middle plane using the chosen settings for the currently triggered channel.
    iii. If the resulting image is not ideal, adjust the exposure time and laser power, then repeat.
      **CRITICAL:** Make sure to move to another region of interest (ROI) after optimizing the imaging settings so the cells have not been excited previously for optimal and reliable results.
  e. Confirm the acquisition sequence.
  f. Name your file with date and strain name and give it a file path in your hard drive.
  g. Run the sequence.
    **CRITICAL:** The image needs to have sufficient signal to noise ratio but cannot have saturated pixels. Saturated pixels will underestimate the fluorescence intensity. Click “Pixel Saturation Indicator” or press “Ctrl + Shift + S”. If the resulting image has highlighted portions, those pixels are saturated, which can be corrected by using a lower laser power and/or shorter exposure time.

### Image analysis

**Timing: ≥1 h**

With the images obtained, the data will be analyzed using different visualization methods to best understand the localization of the epitope-tagged POI. Since this step is solely digital, each step in this process can have a “*Pause Point*” if you can save your work and step away.

5. Open the files in NIS-Elements Advanced Research- Analysis.
  a. “File” -> “Open”, then select your files.

***Note:*** For this analysis, we have used NIS-Elements Advanced Research- Analysis 6.02.01.

***Note:*** We assess the collected images using NIS-Elements Advanced Research- Analysis and Fiji. Using NIS, the 3D projection can be easily made and manipulated. In Fiji, the fluorescence intensity and other parameters can be measured.

6. Adjust the “LUTS” values to see the POI clearly.

***Note:*** Set the minimal LUTS value at the base of the peak and adjust the maximal value to desired contrast and brightness. See troubleshooting 3.

**Figure 3:**
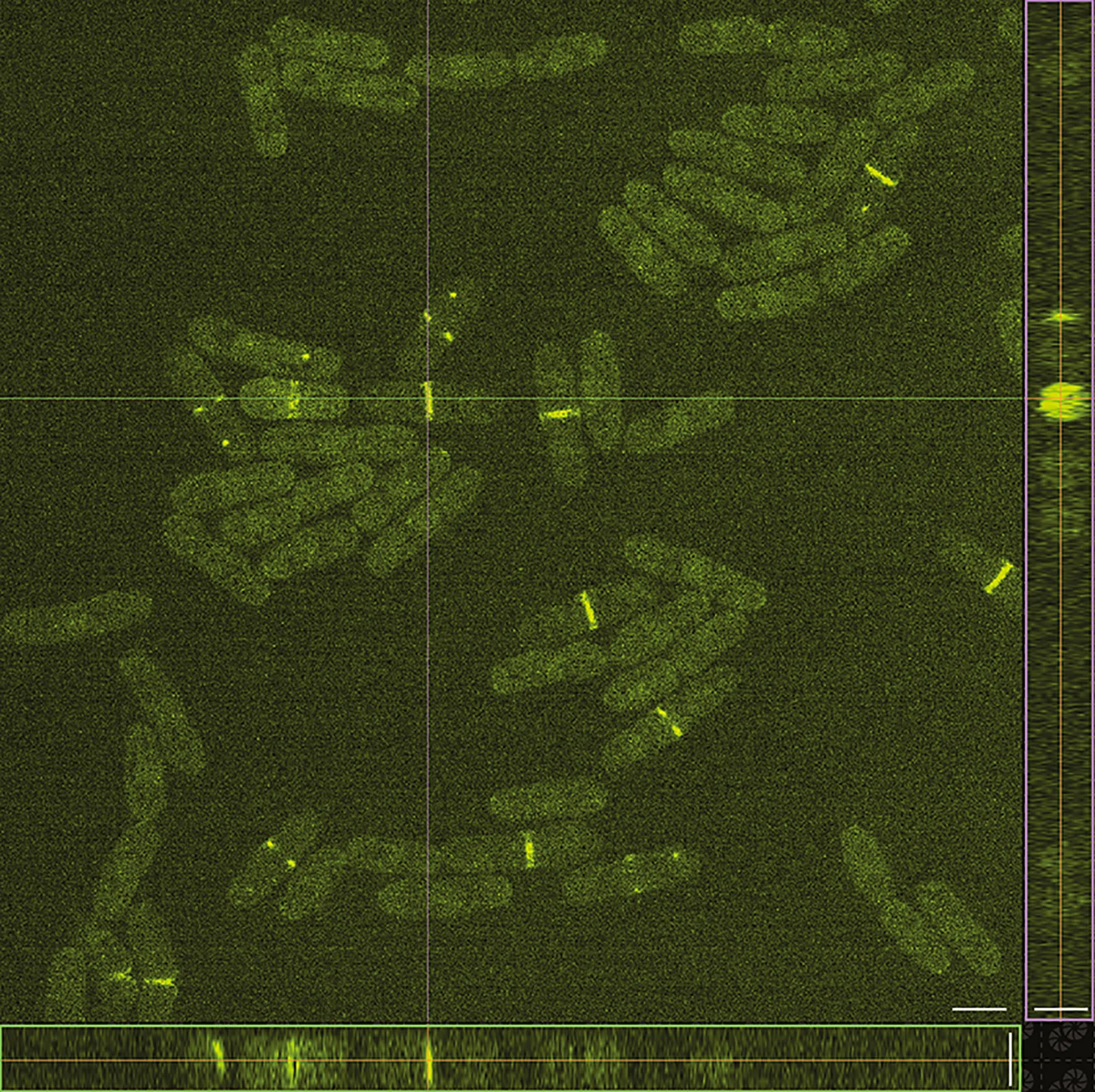
An example of the “Slices View” The “Slices View” for Pmo25-mECitrine shows the Z slices across the two axes in the image, revealing Pmo25 localization at the division site and spindle pole body. The horizontal box at the bottom displays the Z slice image along the green line, and the vertical box on the right displays the Z slices image along the purple line. Scale bars, 5 μm.
  b. Drag the cursor over the division site so that the septum is parallel to either the x or y-axis.
  c. Take a screenshot to obtain an image of the ring.

***Note:*** Figure 8D in Ye, et al. 2025 is an example of using this projection to understand protein distribution at the division site.^1^

***Note:*** It may be necessary to rotate the image so that a cell of particular interest is horizontal or vertical by clicking “Image” -> “Rotate” -> “Rotate By Degree”

7. Assess protein localization at the division plane.
  a. Click “Slices View” to observe the Z-stacks for each XY point (Figure 3).

8. Assess protein localization via a 3D projection.
  a. Click “Volume View” (Figure 4).
    ***Optional:*** It may be helpful to first crop the image to focus on individual cells of interest by clicking “Image” -> “Crop” and then fitting the ROI onto the cell(s) of interest.

**Figure 4:**
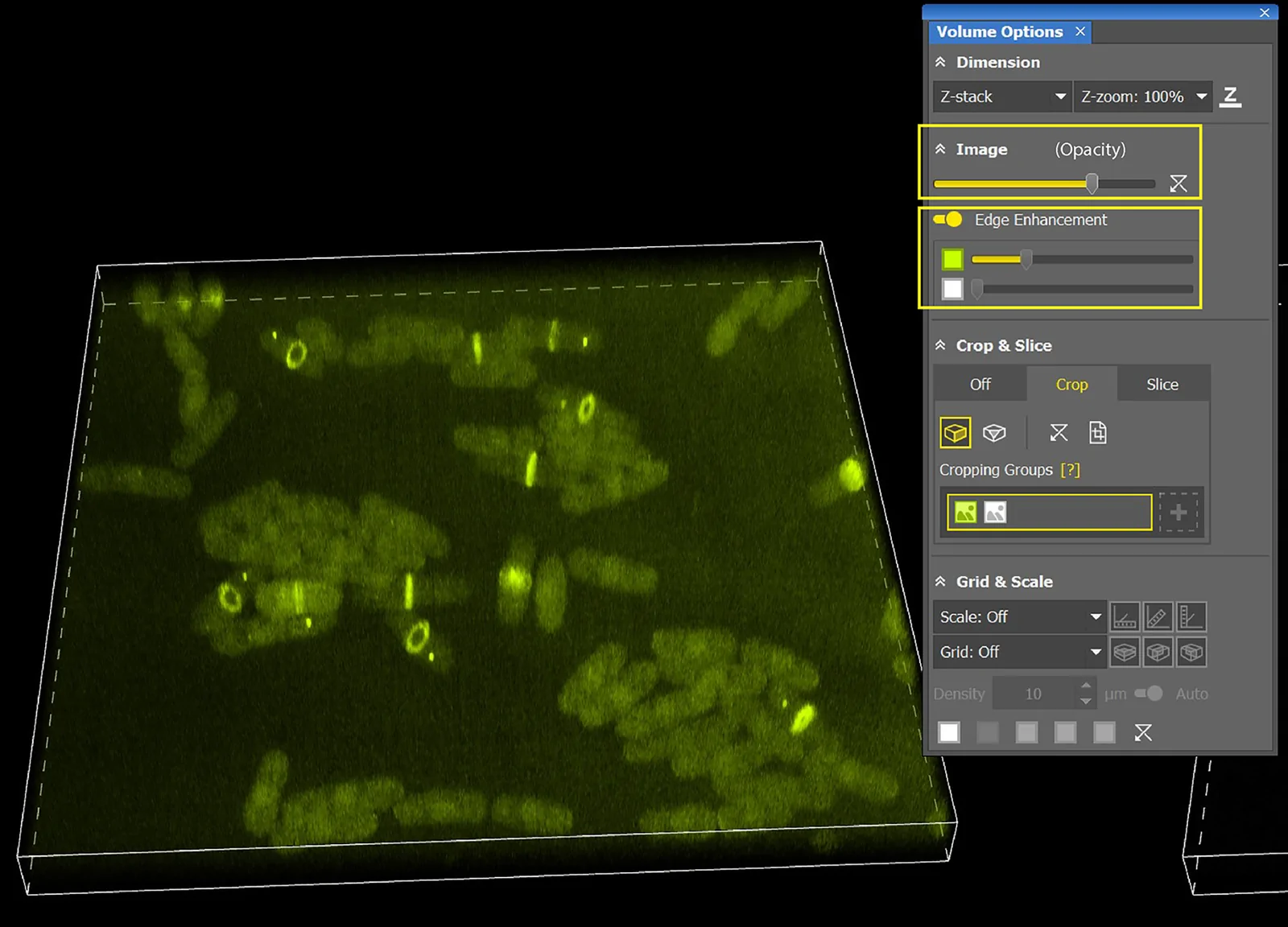
An example of the “Volume View” The “Volume View” showing the 3D projection of Pmo25-mECitrine reveals Pmo25 structures at the spindle pole body and division site. Adjusting the “Volume Options” is necessary to obtain a clear and assessable volumetric projection: the “Opacity” is to modify the image opacity and the “Edge Enhancement” to is smooth the edges for a selected channel, which are highlighted by yellow rectangles. Volumetrix box dimensions, 94 μm × 94 μm × 6 μm.
  b. Adjust the LUTS value to obtain a clear image.
  c. Likewise, adjust the smoothing/rounding of the image by clicking “Volume Options” and adjusting the opacity and the edge enhancement for your channel(s).
    ***Note:*** The visualization of the volumetric projection will take a lot of effort to find the ideal settings that remove the background and provide a clear result. See troubleshooting 4.
  d. Take a screenshot (or screen recording if analyzing a time-lapse movie) to show protein localization at the division site.
9. Measure the fluorescence intensity in Fiji (Figure 5).
  a. Open Fiji and the image file. “File” -> “Open” then click the intended file.
    ***Note:*** For this analysis, we have used Fiji version 1.54k.
  b. Create a SUM or Maximum intensity projection. “Image” -> “Stacks” -> “Z Project” -> “SUM Intensity Projection” or “Maximum Intensity Projection”.
    ***Note:*** We use a SUM intensity projection for most analyses, but a Maximum intensity projection may be used for following the peak intensity.
  c. Open the Color Adjust panel. “Image” -> “Color” -> “Adjust” or simply press “Ctrl + Shift + C”
  d. Adjust the LUTS in the same manner as above. First press “Auto” then adjust.
  e. Draw an ROI around the division site, other locations, or along the cell boundary using either “Polygon ROI” or “Free Hand ROI”.
    ***Note:*** We recommend using the “Polygon ROI” to draw an ROI around the division site signal. “Free Hand ROI” is useful as well if you can draw it accurately.
    **CRITICAL:** It is important to ensure that your ROI captures >95% the division site signal but not needlessly includes either cytoplasmic signal or background signal outside the cell.
  f. Measure the intensity. Press “M”. If it is a time-lapse movie, click “Image” -> “Stacks” -> “Measure Stacks” to obtain the intensity of the ROI at each time point.
  g. Obtain the Mean Intensity (Figure 5).
    ***Note:*** Repeat steps e to g to measure all the cells in the image if needed, then open the next image file if necessary. If the illumination is uniform in the image, we measure all the cells in the image for global intensity in each cell to avoid bias. We recommend to quantify at least 30–50 cells per replicate at the cell-cycle stage that you are analyzing. However, this can be difficult depending on the strain. More details are available in our earlier publications.^1^^,^^13^^,^^14^^,^^16^
    ***Note:*** We use wild-type cells imaged under identical settings to subtract the background for global intensity. For local intensity, 2× concentric ROI outside the original ROI is used to calculate background.

**Figure 5:**
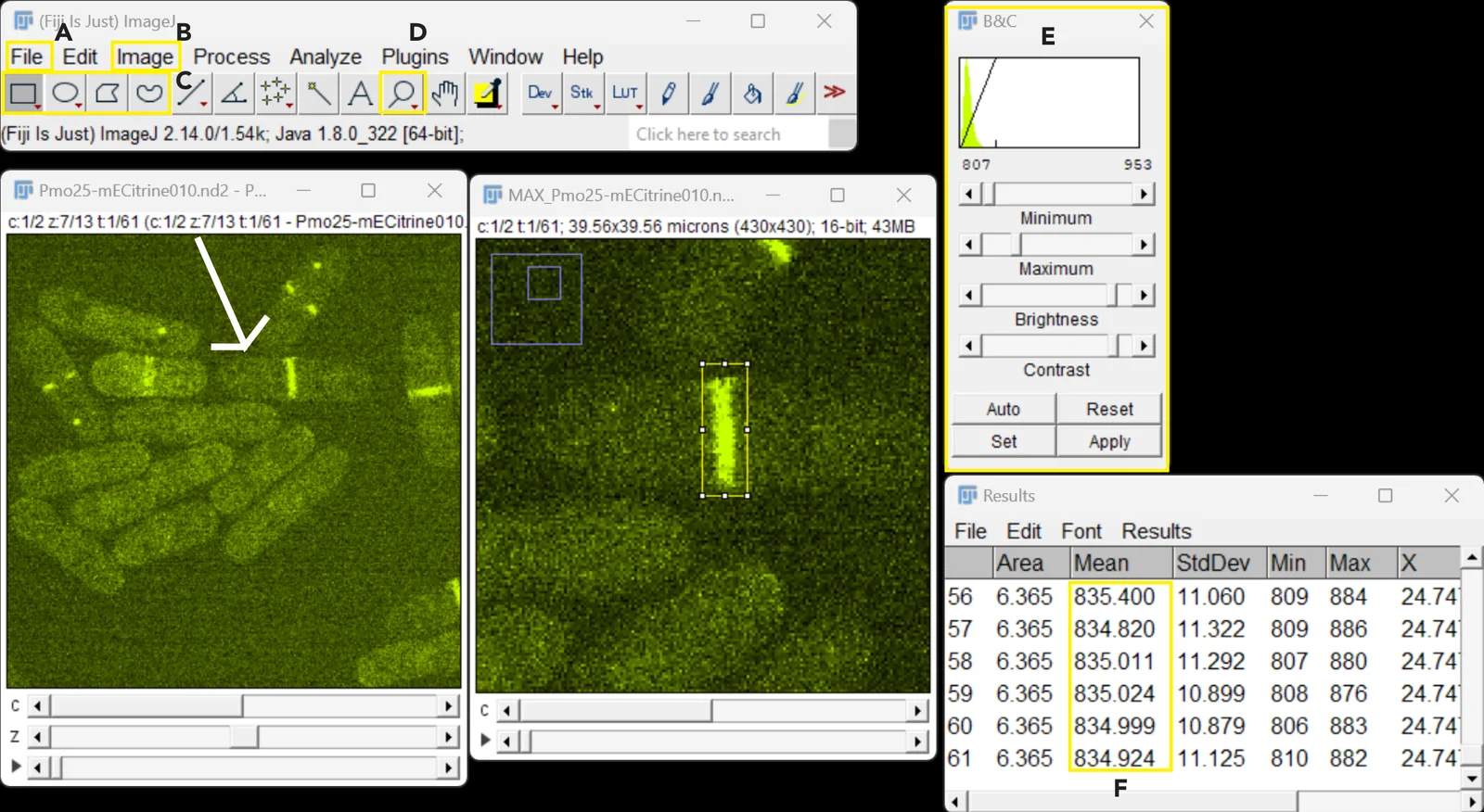
Data analysis setup in Fiji This figure shows the analysis of Pmo25-mECitrine in Fiji. (Left) Opened raw image in Fiji. (Right) Maximum intensity projection of an image zoomed at 200% for one cell of interest (marked by an arrow on the left). Note the ROI drawn around the division site. (A) The “File” tab has options to open and save files and other functions. (B) “Image” tab has options to adjust the channels, colors, image stacks, and others, including making Z projections and measuring the stacks. (C) ROI drawing options from left to right: rectangle, oval, polygon, free hand. (D) Zoom function to zoom in and out. (E) The Brightness & Contrast panel to adjust the display of the pixel intensities. (F) The “Results” window showing the measured results of the selected ROI including mean intensity headed by the “Mean” column.

### Determining protein interactions and total protein concentrations

**Timing: ∼2.5 days**

When the local concentration of a POI differs in wild type and mutant cells, it is necessary to investigate if the difference is due to changes in global protein concentration, which can be caused by variations in protein expression levels or stability. Although it is possible to measure the global concentration using fluorescence intensity,^18^^,^^19^^,^^27^ it cannot reveal if the intensity is from the tagged POI or the tag alone (for example, the free GFP cleaved from the POI may be very stable in cells). Thus, it is necessary to perform Western blotting to examine protein concentration using cell extracts. In addition, the cell extracts can also be used to test protein interactions using Co-Immunoprecipitation (Co-IP). Here, we provide an optimized protocol of Co-IP and Western blotting using *S. pombe* cell extracts, which has been used successfully in Ye, et al. 2025 and other publications.^1^^,^^14^^,^^15^

10. Grow, collect, and lyophilize the cells.
  a. Grow the cells expressing the tagged POI in YE5S liquid medium (or other medium) at a suitable temperature to OD_595_ = ∼0.5.
    ***Note:*** Using >500 mL of cells provides enough cells to allow several IPs.
  b. Harvest the cells by centrifuging at 3,700 × *g* for 5 min. Discard the supernatant and add 5 mL ddH_2_O.
  c. Resuspend the pellet and transfer all the cells to a 50 mL conical-bottom tube and fill the tube with sterile ddH_2_O.
  d. Centrifuge the tube at 3,700 × *g* for 5 min.
  e. Remove the supernatant as much as possible. Use a plastic pipet or a P1000 pipet tip to spread the wet cells along the tube wall.
  f. Replace the cap with a cap with ≥4 holes (Figure 6).
    ***Note:*** A big cell pellet or cluster is harder to dry out during lyophilization.

**Figure 6:**
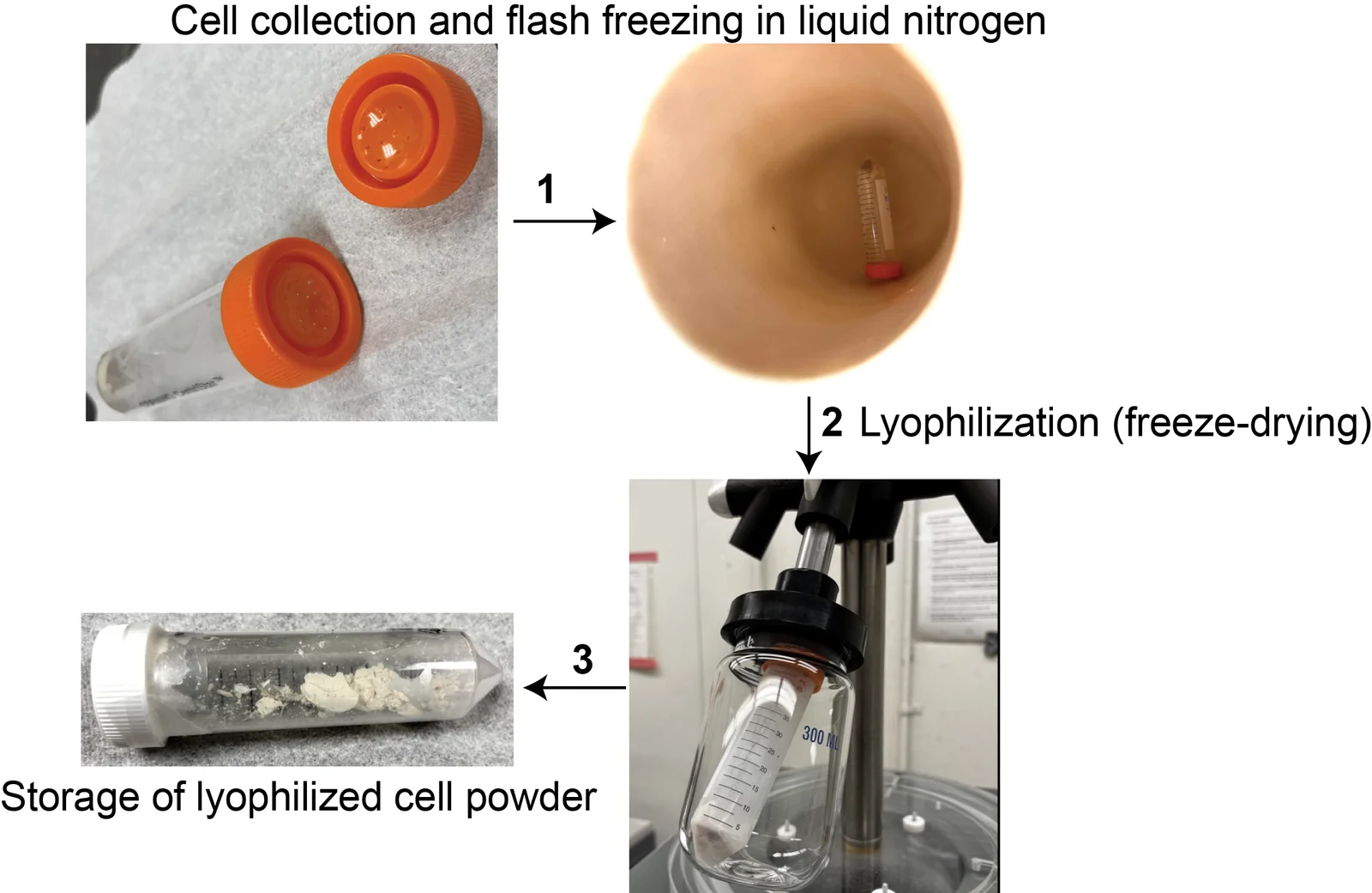
Workflow of sample preparation by lyophilization for CoIP and protein quantification by Western blotting Step 1. Cell collection and flash freezing in liquid nitrogen. Cells are collected in a 50 mL sterile tube by centrifugation at 4°C. The cell pellet is spread along the inner wall of the tube. The tube is then closed with a perforated cap and flash frozen in liquid nitrogen. Step 2. Lyophilization (freeze-drying). The flash-frozen tube is placed inside a lyophilizer glass chamber and sealed with a rubber stopper. The chamber is connected to a pre-cooled lyophilizer. The valve corresponding to the chamber containing the sample is opened, while all other valves remain closed. Samples are lyophilized for ≥24 hr. Step 3. Storage of lyophilized cell powder. After lyophilization, the vacuum is released and the glass chamber is removed. The perforated cap is immediately replaced with the original airtight cap, which is further sealed with parafilm. The dried cell pellets are stored at −80°C until protein extraction for Co-IP and/or Western blot analysis.
  g. Freeze the cells in liquid nitrogen.
    **Pause point:** The cells in liquid nitrogen can be stored at −70/–80°C for up to a week or longer. When you are ready to proceed, simply place the tube from the freezer into liquid nitrogen again and carry it to the lyophilizer.
  h. Put the tube onto a lyophilizer, allow it to dry for 24–48 hr.
  i. Remove the tube from the lyophilizer, cap it with an intact cap without holes. Seal the cap with parafilm and store the tube at −80 or −70°C.
    ***Note:*** The expected mass of the pellet is at least 10 mg per 100 mL of culture.
11. Make cell extracts for IP and Western blotting.
  ***Note:*** Have the centrifuge and rotor ready and cooled at 4°C. Before starting protein purification, have the required amount of IP buffer ready on ice.
  a. Add one tablet of EDTA-free protease inhibitors per 50 mL IP buffer and PMSF (from 0.1 M stock in isopropanol to a final concentration of 1 mM) just before using.
  b. Swiftly weigh 30–100 mg of lyophilized cells for each strain (this amount can vary according to the protein abundance in cells) and place in a mortar.
  c. Grind the cells with a pestle to a fine powder.
  d. Use a small piece of weighing paper to scrape and transfer the cell powder from the mortar to one labelled round-bottom 1.5 mL low adhesion centrifuge tube.
    **CRITICAL:** Keep the cells as dry as possible during the weighing and grinding because they become hygroscopic after lyophilization. Lyophilized cells burst and lyse when moistened. If cells lyse without the IP buffer, the POI can be degraded by proteases. See troubleshooting 5.
  e. Add ice cold IP buffer to cell powder at a ratio of 10 μL of buffer per 1 mg of lyophilized cell powder.
    ***Note:*** Keep the tubes on ice before all the samples are ready.
    ***Note:*** The lysate is too viscous to handle if less buffer is added.
  f. Resuspend and mix the cells with the IP buffer by inverting or using a pipet tip (solution is very viscous, be cautious not to block the pipette tip).
  g. Once all the samples are in IP buffer, vortex at the highest setting 3–4 times for 5–10 sec for each sample. Place the tubes on ice between vortexing.
    **CRITICAL:** Rigorous pipetting up and down causes foaming and protein loss so it is best to gently mix by stirring using a pipet tip.
  h. Spin at 845 × *g* for 2 min at 4°C.
  i. Transfer the supernatant to a fresh ice-cold low adhesion microcentrifuge tube without disturbing the pellet.
    ***Note:*** Since the pellet is loose, it is fine if a small amount of pellet is picked up at this step.
  j. Spin again at 21,130 × *g* for 10 min at 4°C.
  k. Transfer the supernatant to another ice-cold low adhesion microcentrifuge tube. Do not disturb the lipid layer on top of the lysate or pick up the pellet.
    ***Note:*** Remember to save about 12–15 μL of the cell extract to be used as the pre-IP samples.
    ***Note:*** It is possible to store the cell lysate at −80°C after flash freezing with liquid nitrogen. However, protein degradation will still happen. Especially for low abundant proteins, it is better to use fresh cell lysate to test the protein interactions and to quantify protein levels.
12. Co-Immunoprecipitation (Co-IP).
  ***Note:*** This procedure is specifically for pulling down GFP or YFP labelled proteins from cell lysates. We use magnetic Protein-G Dynabeads to bind the GFP antibody. Other antibodies (such as anti-Myc or anti-RFP) can be used as well for Co-IP.
  a. Resuspend the Protein-G covalently coupled magnetic Dynabeads to make it uniform. For each sample, pipet 60 μL beads to a low adhesion microcentrifuge tube.
  b. Wash 3 times with 1 mL ice-cold PBS for each wash. Tap the microtube to resuspend the beads during each wash before collecting cells with the magnetic rack.
  c. Resuspend in 600 μL PBS, add 5 μg antibody (different antibodies might have different concentrations and thus the volumes used might be different) and incubate on a nutator at 22–24°C for 1 hr.
  d. During the incubation, tap the tubes to keep the beads resuspended from time to time.
    ***Note:*** During the incubation, follow step 11 to prepare the lysate. When the lysate is ready, proceed to step e below.
  e. Collect the beads using the magnetic rack.
  f. Remove the supernatant and wash the beads 3 times with 1 mL cold PBS for each wash.
  g. Wash once with 1 mL coldIP buffer. Keep the tube on ice.
  h. Collect the beads using the magnetic rack and remove all the supernatant including any bubbles in the tube.
  i. Add all the supernatant from step 11k except the 12–15 μL pre-IP sample to the equilibrated beads and resuspend the beads by tapping.
  j. Incubate for 90 min at 4°C on the nutator.
  k. During the incubation, tap the tube and resuspend the beads every 20 min or so if possible.
  l. During the incubation, spin down the leftover lysate at 4°C using the “short” function on a microcentrifuge.
  m. Transfer 4 to 12 μL (the volume depends on the abundance of POI) to another tube and add 1–3 μL 5× Laemmli sample buffer.
  n. Add another 40 μL 1× Laemmli sample buffer to match the final volume of the IP sample.
  o. Boil for 5 min, and spin down using the “short” function on a microcentrifuge. **This is the pre-IP sample.**
    ***Note:*** Pressing the “short” button on the centrifuge for several seconds should be enough; this is just to spin down the buffer that evaporated to the top of the tube during boiling.
    ***Note:*** Because of the viscosity of the lysate, it is hard to get 12 μL leftover, and it varies from sample to sample. Therefore, picking up the same volume of lysates between different samples and transferring to another tube before adding the sample buffer is very important.
  p. Collect the beads using the magnetic rack and remove the supernatant.
  q. Wash 5 times with 1 mL cold wash buffer each time: 2x with wash buffer I and 3x with wash buffer II. During each wash, resuspend the beads and rotate for 1 min on a nutator for mixing beads with buffer before collecting the beads using the magnetic rack.
    ***Note:*** Usually wash steps are performed at 22–24°C, but in the case of some sensitive interactions, doing it in a cold room (4°C) might be beneficial.
  r. Remove all of the supernatant including the bubbles.
  s. Add 50 μL 1× Laemmli sample buffer to the beads and resuspend by pipetting to elute the proteins.
  t. Boil for 5 min and spin down using the “short” function on a microcentrifuge.
  u. Use the magnetic rack to separate beads and supernatant. Transfer all the supernatant to a new tube: **this is the IP sample.**
13. Western blotting.
  a. Use a 4–20% gradient SDS-PAGE with 10 wells (30 μL/well capacity). Put the gel in the gel tank following the manufacturer’s instructions. Remember to remove the tape at the bottom of the gel before putting it in the tank otherwise current will not flow.
    ***Note:*** Make sure that the running buffer is not leaking from the cassette before loading the samples.
    ***Note:*** For larger molecular weight proteins (∼200 kDa), a lower % SDS-PAGE will allow for better resolution.
  b. Load 25 μL sample (to avoid overflow to nearby wells) using long gel loading tips to each well.
    ***Note:*** The wells on the edge of gel can be avoided if number of samples are less than total number of wells.
    ***Note:*** Load the same volume of 1× Laemmli sample buffer to the empty lanes, which will help the proteins run on straight lines instead spreading to the empty lanes.
  c. Load 10–15 μL pre-stained protein marker.
  d. Run the gel at 100 V until the dye front reaches the bottom.
  e. Open the gel from the caster plates per the manufacturer’s instructions, remove the stacking gel (upper gel), and make a small cut on the corner to mark the direction of samples.
    Prepare the transfer buffer and cool it at 4°C.
    ***Note:*** You can make transfer buffer from 10× stock but keep in mind to add 20% methanol just before using. Ethanol can also be used.
  f. Cut 4 gel sized Whatman filter papers and 1 piece of PVDF membrane (nitrocellulose membrane can also be used).
  g. Add methanol to soak the PVDF membrane for 1 min.
  h. Pour out the methanol and rinse the PVDF membrane once in transfer buffer and leave it to equilibrate in transfer buffer.
  i. Put the Whatman filter paper, sponge, and gel in transfer buffer for ≥10 min.
  j. Setup the sandwich in the transfer buffer, avoid air bubbles.
  k. Add an ice pack to the transfer tank, and transfer the proteins for 1 hr at 100 V at 4°C.
    ***Note:*** To keep the transfer tanks at 4°C, we surround the transfer tank with ice in a Styrofoam box.
    *Alternatives*: Transfer can be done at 30 V (∼12–16 hr) at 4°C.
  l. Prepare the blocking solution before the transfer is done.
    ***Note:*** Blocking is efficient with either 3% BSA or 5% non fat dry milk in 1× TBST.
    ***Note:*** Carnation milk has to be completely dissolved in TBST at 22–24°C for ≥30 min while stirring. Otherwise, the blot will have uneven background when developed.
  m. Remove the membrane from the sandwich carefully after transfer and cut one corner same as the gel to preserve the orientation.
  n. Soak the membrane in 10 mL of blocking solution at 22–24°C for 1 hr on a gel rocker.
    ***Note:*** The pre-stained protein marker is visible on the membrane after transfer.
    *Alternatives:* The membrane can also be blocked for 12–16 hr at 4°C.
  o. After blocking, incubate the membrane with primary antibodies against GFP (1:1,000) and tubulin (TAT1, 1:10,000) in 1× TBST buffer with 1% carnation milk for 1 hr at 22–24°C while shaking on a rocker.
    *Alternatives*: Tubulin is used as a loading control. Other proteins with a constant concentration during the cell cycle can also be used, such as actin, Cdc2, etc.
  p. Wash the membrane in 10–15 mL of TBST buffer three times quickly, then one additional time for 5 min on a rocker and one more time for 10 min on a rocker at 22–24°C. Change the TBST buffer on every wash step.
    ***Note:*** Keep the transferred protein faceup on the PVDF membrane so that you do not lose protein by rubbing against the bottom of the container.
  q. Incubate the membrane with the secondary antibody conjugated with HRP against the source of primary antibody for 1 hr at 22–24°C while shaking on a rocker. Prepare 10 mL of the antibody solution (1:5,000) in TBST with 1% carnation milk.
  r. Wash the membrane in the same manner as step p.
  s. Develop the signal using the ECL kit and follow the manufacturer’s instructions.
  t. Image the developed blot on an imager quickly and transfer the data files for further analysis.
    ***Note:*** From this step onward, we will describe image analysis using software for our imager (iBright CL1500), but these steps can be easily adjusted for whichever imager you utilize. Fiji can also be used. See troubleshooting 6.
    **Pause point:** Since the following steps are digital, you can stop, save your work, and walk away during this process.
14. Western Image analysis and quantifications.
  a. Download and install the software for image analysis from the provider whose imager has been used for detection.
  b. Click “Import” to open the g2i file needed for analysis. The images will be available in the gallery from where you select the appropriate images, then hit “Next” on the bottom right corner.
    ***Note:*** The software will automatically identify the lanes and bands.
  c. For manually adjusting/adding/deleting the lanes, use “Edit Layers” panel on right hand side (select a lane and hit + or – icon to add or delete, use skew icon to adjust the direction of lane; similarly use + or – icon to add or delete a band).
    ***Optional:*** You can also manually adjust the brightness and contrast from the “View & Edit channels” menu on the right side.
  d. Once finalized, click “Apply” on the center bottom of the screen.
  e. To generate a report of the current analysis after finalizing the bands, go to the “Export analysis report” tab on the top right corner.
  f. From the report, find “Lane and band analysis data table” near the end for further statistics.
  g. Use “Local Bg. Corr. Vol” or “Rolling Bg. Corr. Vol” values based on the requirement and your preferences.
    ***Note:*** If you have multiple blots on the same image, it will generate a single report having multiple blots designated as numbers of frames. It will also show the intensity values for both the Membrane and Chemi channels depending on what you selected when finalizing the bands.

## Expected outcomes

**Figure 7:**
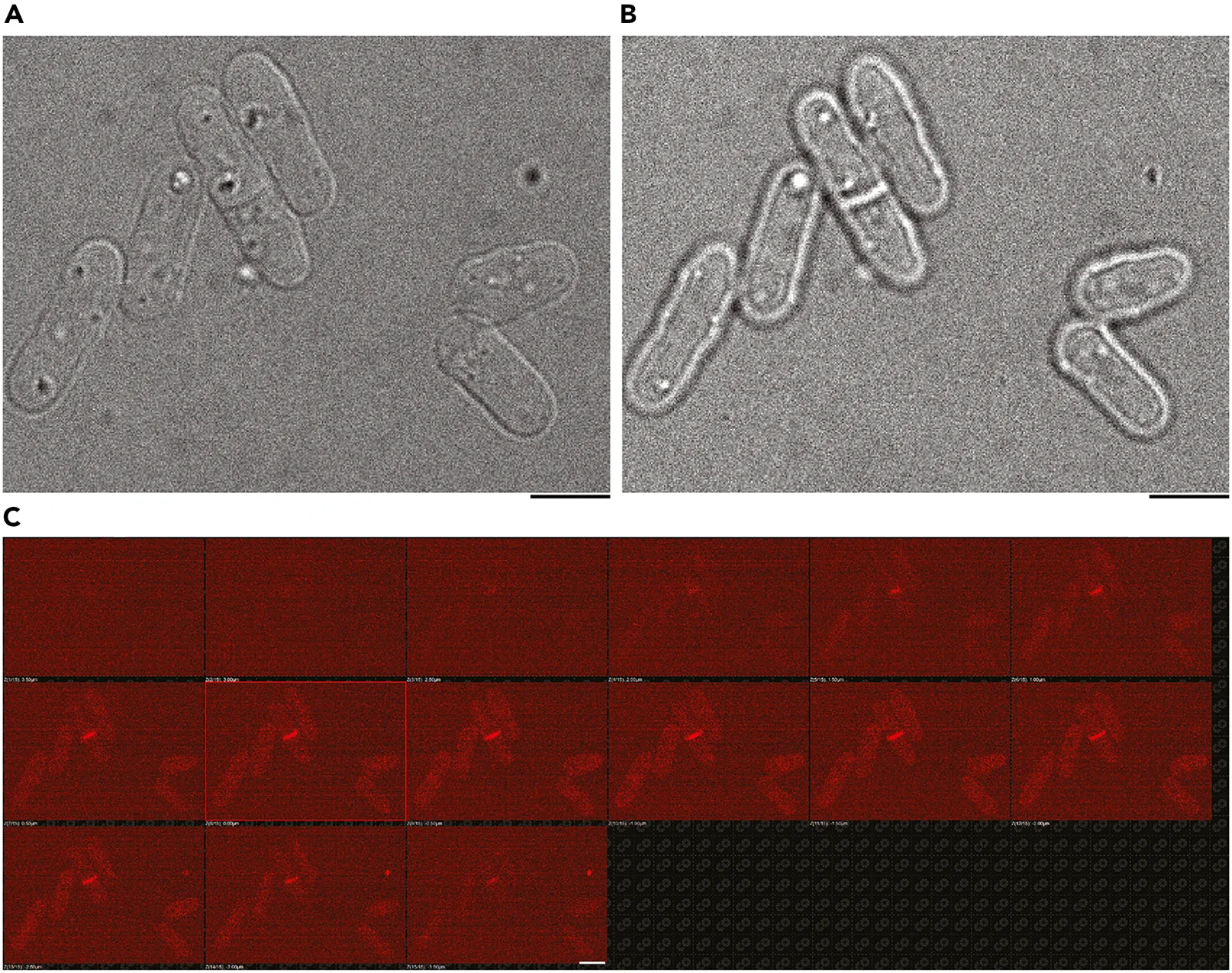
An example of out of focus images Images of Pmo25-tdTomato (JW7944) on confocal microscopy. (A) Example of cells that are in-focus in the DIC channel. (B) Example of cells that are out of focus in the DIC channel. (C) Example of the Z-stack imaged while the cells were out of focus. The early Z slices have no signals, but the last slice still misses some signals. Scale bars, 5 μm.

This protocol, when completed, provides multiple reliable avenues for obtaining images and data. First, the localization (and potentially the kinetics) of the POI at the division site or other locations of interest can be determined. In Ye, et al. 2025, Figures 1A and 1B shows how the timing of contractile-ring proteins were reliably assessed using time-course imaging and a cell cycle marker; Figures 4B–4E and 7A–7D show the changes in protein intensity over time and the peak intensity changes in mutant cells; and Figure 5 shows an example of assessing the protein kinetics without time-course videos due to low expression levels. Second, this protocol allows to test protein interactions by Co-IPs and to measure total protein concentration by Western blotting using the lyophilization process, which is very efficient in obtaining cell extracts. In Figures 4F, 4G, and 7E; and Figures 9F and 9G of Ye, et al. 2025, this procedure was successfully carry out to obtain protein concentrations and validate the previous results.^1^

## Limitations

This protocol effectively describes how to obtain key quantitative information about a POI in *S. pombe*, especially at the division site. Although the protocol is applicable in general to other model organisms, the gene targeting can be quite different if homologous recombination is not efficient there. In addition, the 3′ UTR is replaced with the *ADH1* terminator, which may affect the gene expression level. Moreover, cell growth, preparation, and imaging could vary dramatically. This protocol, when conducted in different mutant backgrounds, can provide crucial information of a protein’s interactions; however, this is not sufficient for understanding a complex mechanism of actions. Further genetic and biochemical interaction studies are required. Lastly, the quality of the results depends on the microscope being used, as a microscope with lower resolution will limit the possible analyses greatly.

## Troubleshooting

### Problem 1

The PCR for amplifying the DNA for gene targeting failed (related to “before you begin” step 2a-c).

### Potential solution

• It is possible that the GC content of the primer or the template DNA (such as natMX6) is too high. We recommend adding 5% DMSO to the PCR reaction mixture.
• If the band is smeared or smaller than expected, make sure the extension time is long enough for the expected product, normally 1 min/kb.
• Lastly, ensure that the annealing temperature is correctly calculated based on the 20 nt of homology between the primers and the plasmid DNA.

### Problem 2

The transformants from the gene targeting with an epitope tag are unattainable or grown defectively (related to “before you begin” step 3).

### Potential solution

• The fluorescent or other epitope tag being used likely causes a loss of function of the POI. If your POI is essential, a loss of functionality induced by the tag could lead to no surviving transformants. We recommend trying several different epitope tags at the same time. For example, in our experience, tdTomato and 3HA tags, but not mEGFP or mYFP, compromise septin localization and function.^13^
• Flexible linkers rich in amino acids such as glycine and serine can be inserted between POI and the tag to make a more functional fusion protein.^13^
• Consider N-terminal^4^^,^^10^^,^^28^ or internal tagging^29^ if the C-terminus cannot be functionally tagged. You could either embed your cassette with a new promoter for your POI (which will alter expression levels)^28^^,^^30^ or utilize the *Delitto Perfecto* method^31^ to use counter selection to remove the selectable marker and restore the native promoter or cloning the endogenous promoter to add the fluorescent tag without displacing the promoter.

### Problem 3

The fluorescence images do not have enough signal to noise or contrast (related to step 6).

### Potential solution

• To reduce the background autofluorescence intensity for low abundance proteins, you can switch the growth media from the rich YE5S medium to the minimal EMM5S medium, which has much lower autofluorescence.
• Another possibility is that the imaging conditions in Step 4 are not optimal. It may be necessary to increase the exposure time and/or the laser power for imaging. However, the downside is that the fluorescence signal may photobleach more quickly. An appropriate trade-off is needed. Depending on the camera, gain and binning can be used to increase the signal, but the binning reduces spatial resolution.

### Problem 4

The 3D projection of fluorescence images is fuzzy or incomplete. This could be because of poor image resolution, missing image slices, and/or the signal loss from photobleaching (related to step 8).

### Potential solution

• It is possible that not enough images were taken in the Z focal-plane in Step 4. As a rule of thumb, the Nyquist-Shannon sampling theorem^26^ is a starting point:

Z step size = 0.8*λn*/(NA),^2^ *where* λ *= emission wavelength, n = refractive index, NA = Numerical Aperture of the objective.*

The slice spacing could be adjusted according to the signal strength and experimental purpose.

• It is also possible that your imaging range in the Z axis was incorrect in Step 4 if the 3D projection is cutting off the top or bottom of the cell. This means you would need to increase the “range” value in the “Z” section for image acquisition. For some mutants with an increased cell diameter, such as *pmo25* mutants in Ye, et al. 2025, the range will likely need to be increased.^1^
• Similarly, it is possible that your cells are not centered at the central focal plane in Step 4. Figure 7 shows “centered” vs “off centered” DIC images and the slice view that would result from that.
• If the laser power and/or the exposure time in step 4 is more than necessary, the signal can be saturated in certain Z slices or bleed into the other slices.

### Problem 5

Cells are not dry enough after lyophilization, which makes it harder to break the cells for Co-IP and Western blotting (related to step 11).

### Potential solution

• It is likely that the cell pellet was not frozen properly in liquid nitrogen or warmed up during setting up the lyophilizer. We recommend flash freezing the pellet again in liquid nitrogen for 2–5 min and then putting the tube back onto the lyophilizer.
• Another reason could be that you forgot to replace the tube cap with a cap that has holes in it. In this case the liquid will not evaporate from the tube and the pellet will not dry out.
• Ensure that all the openings of the lyophilizer are closed. Otherwise, the vacuum pump will fail to make a vacuum, and the samples will not dry out.

### Problem 6

There was no band present after the Co-IP (related to step 13).

### Potential solution

• There could be many reasons that no bands are visible in Co-IP samples after immunoprecipitation. If you see protein bands in the Pre-IP samples but not in the Co-IP sample, then there might be no interaction between the proteins that you are testing. If the protein bands in pre-IP are too weak, increase the amount of dried cell powder for the cell extract to increase the protein concentration in the Co-IP samples.
• If you see no pre-stained marker bands after transfer, check if you made the transfer sandwich correctly and put the sandwich in gel tank in the correct orientation. If you transfer the proteins for too long or at too high voltage, the proteins may transfer and migrate through the PVDF or nitrocellulose membrane, especially for low molecular weight proteins.
• We recommend increasing the concentration of the primary antibody in case the antibody is too diluted.

## Resource availability

### Lead contact

Further information and requests for resources and reagents should be directed to and will be fulfilled by the lead contact, Jian-Qiu Wu (wu.620@osu.edu).

### Technical contact

Technical questions on executing this protocol should be directed to and will be answered by the technical contact, Jack Gregory (gregory.600@osu.edu).

### Materials availability

This study did not generate new unique reagents.

### Data and code availability

This study did not generate/analyze any new datasets or codes.

## Acknowledgments

We thank the former members of our laboratory for developing and optimizing the protocols and methods described in this article: Valerie Coffman, I-Ju Lee, Ning Wang, Reshma Davidson, Yajun Liu, Yi-hua Zhu, Kenneth Gerien, Sha Zhang, and Nicholas Ricottilli. J.R.G. was supported by the Cellular, Molecular, and Biochemical Sciences Training Program (T32 GM141955) from the National Institutes of Health. D.S. was supported by the Postdoctoral Candidate Pelotonia Scholars Program of The Ohio State University. The work was supported by the National Institute of General Medical Sciences of the National Institutes of Health (grants R01GM118746 and R35GM164259 to J.-Q.W.). The content is solely the responsibility of the authors and does not necessarily represent the official views of The Ohio State University or the National Institutes of Health.

## Author contributions

Conceptualization, J.R.G. and J.-Q.W.; investigation, J.R.G.; methodology, J.R.G., Y.Y., D.S., A.H.O., and J.-Q.W.; validation, J.R.G., Y.Y., D.S., A.H.O., and J.-Q.W.; resources, J.-Q.W.; supervision, J.-Q.W.; writing – original draft, J.R.G.; writing – review and editing, J.R.G., Y.Y., D.S., A.H.O., and J.-Q.W.

## Declaration of interests

The authors declare no competing interests.
